# *Akkermansia muciniphila* supplementation prevents cognitive impairment in sleep-deprived mice by modulating microglial engulfment of synapses

**DOI:** 10.1080/19490976.2023.2252764

**Published:** 2023-09-06

**Authors:** Na Li, Shuwen Tan, Yue Wang, Jiao Deng, Nan Wang, Shan Zhu, Wen Tian, Jing Xu, Qiang Wang

**Affiliations:** a Department of Anesthesiology & Center for Brain Science, The First Affiliated Hospital of Xi’an Jiaotong University, Xi’an, Shaanxi, China; b Department of Anesthesiology, Honghui Hospital, Xi’an Jiaotong University, Xi’an, China; c Department of Anesthesiology and Perioperative Medicine and Department of Critical Care Medicine, Xijing Hospital, Fourth Military Medical University, Xi’an, Shaanxi, China; d Department of Pharmacy, No. 95829 Military Hospital of PLA, Wuhan, Hubei, China

**Keywords:** Sleep deprivation, cognitive dysfunction, microglia, synapse, microbiota-gut-brain axis, *Akkermansia muciniphila*, short chain fatty acids

## Abstract

The microbiome-gut-brain axis plays a crucial role in many neurological diseases, including mild cognitive impairment. Sleep deprivation (SD) induces cognitive decline accompanied by alterations in the gut microbiota. However, the role of gut microbiota alterations in SD-induced cognitive dysfunction and the underlying mechanisms remain unclear. Here, we found that dysbiosis of the gut microbiota following pretreatment with broad-spectrum antibiotics worsens SD-induced cognitive impairment in mice. Fecal microbiota transplantation from SD mice to healthy mice induced cognitive impairment. Additionally, the abundance of *Akkermansia muciniphila* (*A. muciniphila*) in the mouse gut microbiota was significantly reduced after 7 days of SD. *A. muciniphila* pretreatment alleviated cognitive dysfunction and prevented synaptic reduction in the hippocampus in SD mice. *A. muciniphila* pretreatment inhibited extensive microglial activation and synaptic engulfment in the hippocampus of SD mice. Metabolomics analysis revealed that *A. muciniphila* pretreatment increased the serum acetate and butanoic acid levels in SD mice. Finally, pretreatment with short-chain fatty acids (SCFAs) inhibited microglial synaptic engulfment and prevented neuronal synaptic loss in SD mice and primary microglia-neuron co-culture following LPS stimulation. Together, our findings illustrate that gut dysbiosis plays an essential role in SD-induced cognitive impairment by activating microglial engulfment at synapses. *A. muciniphila* supplementation may be a novel preventative strategy for SD-induced cognitive dysfunction, by increasing SCFAs production and maintaining microglial homeostasis.

## Introduction

Sleep disorders are common among adults in modern society. Lacking restorative sleep introduces detrimental effects on the brain and bodily function.^1^ The animal model for sleep insufficiency is defined as sleep deprivation (SD). Accumulated evidence suggests that SD may induce cognitive deterioration, such as loss of attention, memory impairment, cognitive decline,^1–3^ even an increased risk of dementia.^4^ However, the underlying mechanism for SD-induced cognitive dysfunction as well as effective, clinically available therapy is still lacking.

Recent studies have shown that sleep insufficiency/deprivation alters the composition of gut microbiota in both humans and rodents.^5,6^ Growing evidence suggests that the gut microbiota – brain axis plays a crucial role in acute and chronic brain diseases,^7,8^ which affect host behavior, such as cognitive dysfunction,^9,10^ depression, and anxiety-related responses.^11,12^ Better sleep quality might be associated with better cognitive performance in healthy older adults with higher proportions of *Verrucomicrobia* and *Lentisphaerae* in the gut microbial.^13^ Gut dysbiosis contributes to both peripheral and central inflammation and impaired cognition induced by SD.^14^ However, the mechanisms underlying these phenomena remain underexplored.

SD may result in deregulated immune responses with increased pro-inflammatory signaling.^15^ Microglia, a major cellular component in the innate immune system of the central nervous system (CNS), actively respond to injury, infection and neuroinflammation.^16^ Microglia engulf synapses and mediate forgetting in the hippocampus of healthy adult mice.^17^ Chronic sleep restriction but not acute sleep loss activates microglia phagocytosis of synaptic elements without obvious signs of inflammation in the cerebrospinal fluid.^18^ Furthermore, inhibition of the microglia activation improves the hippocampus-dependent spatial memory in rats during 48-h SD.^19^ However, the triggers for microglia activation, which could be therapeutic targets in sleep-deprived mice, are still unknown. Recent studies have pointed out that gut microbiota mediates microglial activation under multiple pathological conditions, including Alzheimer’s Disease.^20^ Despite the connection between gut microbiota change and microglia activation in SD mice has been established.^14^ However, the underlying mechanistic underpinnings mediating these changes remain elusive.

This study aimed to investigate the role of gut microbiota alterations and cognitive dysfunction in sleep-deprived mice, as well as the role of microglia in this process. We assessed the effect of antibiotic treatment on SD-induced cognitive impairment and the cognitive function in normal mice that received fecal microbiota transplantation (FMT) from SD mice. We then performed 16S rDNA gene sequencing and qPCR to assess the composition of the gut microbiota and to confirm the quantity change in *Akkermansia muciniphila* (*A. muciniphila*, AKK) in SD mice. Next, we examined the effects of *A. muciniphila* on cognitive impairment, microglial phagocytic activity, and hippocampal synaptic reduction. Furthermore, gut microbiome-associated metabolites were measured after *A. muciniphila* supplementation in mice to identify the targets responsible for microglial activation. Finally, we investigated whether short-chain fatty acids (SCFAs) inhibit synaptic engulfment of microglia and prevent synapse loss *in vivo* and *in vitro*.

## Materials and methods

### Animals and treatments

The animal experiments were approved by the Institutional Animal Care and Use Committee of Xi’an Jiaotong University (Xi’an, China). Male C57BL/6J mice were purchased from Charles River (Beijing, China) and maintained in a specific pathogen-free environment with a strict 24-h reverse light-dark cycle (lights turned on from 8:00 to 20:00). The mice were fed sterile water and commercial standard feed (GB14924.3, China) ad libitum.

Antibiotic treatment started when mice were five weeks old. An antibiotic cocktail^21^ was administered by oral gavage for 14 consecutive days (50 mg/kg vancomycin, 100 mg/kg neomycin, 100 mg/kg metronidazole, and 1 mg/kg amphotericin B). Ampicillin was provided in drinking water (1 g/L).

The SD mouse model was developed using a propeller-based automatic SD system (XR-XS108, Shanghai Xin Ruan) (Supplementary Figure S1a). The system contained a cage in which mice could feed freely while a clockwise-counterclockwise rotating bar was set rotating at the bottom of the cage, 20:00 until 16:00 the next day at 5 rpm, preventing the mice from sleeping. There are 3 turns clockwise and counterclockwise and 18-s intervals every 6 rotations for mice to achieve food and water when needed. The subjects were sleep-deprived for 20 h every day for seven consecutive days (Supplementary Figure S1b). Control (Con) animals were placed in the same cage without a rotating bar to disturb their sleep.

### Fecal microbiota transplantation

Before transplantation, the mice were treated with the antibiotic cocktail for 14 consecutive days, as previously described. Thereafter, mice were given 200 µL of the microbiota suspension from either the sleep-deprived or control mice. Three times a week (FMT was administered on days 1^st^, 3^rd^ and 5^th^) for 3 weeks, starting 48 h after the last gavage of the antibiotics. The microbiota suspension preparation was based on the method described in previous studies.^22,23^ Mice in the empty transplant group received the same antibiotic treatment and were transplanted only with reduced PBS.

### Behavioral tests

Behavioral tests were conducted 7 days after SD or 24 h after the last session of FMT. Performance was tracked and evaluated using a video tracking system (SMART 3.0; Panlab Harvard apparatus). The open-field test (OFT) and elevated plus maze (EPM) tests were used to assess the anxiolytic properties of mice (Supplementary Figure S2a-b), and the novel object recognition (NOR) and Y-maze tests were performed to examine recognition memory and spontaneous rodent behaviors (Supplementary Figure S2c-d). Detailed information is provided in the supplementary material.

### Quantification of bacterial DNA in mice feces

Fecal samples were collected at four key times to identify the effectiveness of antibiotic treatment, including the first day without any treatment, 14 days after antibiotic treatment, 7 days after SD, and the day after all behavioral tests were completed. The samples were immediately frozen at −80°C until DNA extraction. After stool samples were weighed, total genomic DNA was extracted from feces using the Stool DNA Kit (Omega, D4015, China) following the manufacturer’s instructions. DNA quantity was determined fluorometrically using a dsDNA Broad Range-Fluorescence Quantification Assay with a T20 Fluorometer (Life Real, China).

### 16S rDNA sequencing

Fecal samples for the 16S sequencing were collected from mice, either with normal sleep or 7 days after SD. 16S rDNA gene sequencing was performed on the Illumina sequencing platform in a Biotechnology company (Beijing, China). Briefly, PCR amplification was performed spanning the V3-V4 region of the 16S rDNA using primers 341F/806 R (forward 5′-ACTCCTACGGGAGGCAGCAG-3′ and reverse 5′-GGACTACHVGGGTWTCTAAT-3′). Subsequently, sequencing was performed on a MiSeq platform (Illumina) using a 2 × 300 bp-end protocol.

### Quantification of A. muciniphila in cecal content

Cecal contents were collected on the day after all behavioral tests were completed. Total genomic DNA was extracted from cecal contents using the Stool DNA Kit (Omega, D4015, China) as described above. After diluting the DNA templates to 1 ng/µL using RNase-Free Distilled Water, each sample was prepared in triplicate using 2 µL (2 ng of the total DNA template). After diluting the DNA templates to 1 ng/µL with RNase-Free Distilled Water, 2 µL (2 ng of total DNA template) of each sample was loaded in triplicates. The forward primer 5′-CAGCACGTGAAGGTGGGGAC-3′ and reverse primer 5′ -CCTTGCGGTTGGCTTCAGAT-3′^24^ were premixed with SYBR Green Nucleic Acid Gel Stains (SYBR Green) Premix (AG11701, Accurate Biology, China) according to the manufacturer’s protocol. Real-time PCR (qPCR) was performed using a Bio-Rad CFX96 thermal cycler (Bio-Rad, Hercules, California, USA). Reaction mixtures (20 µL total volume) were held at 95°C for 30 s, followed by 40 cycles of 95°C for 5 s and 60°C for 30 s. Melting curve analysis was conducted to confirm the specificity of amplification. Plasmid DNA (gene sequence shown in the supplementary materials), including the corresponding conservative sequence of *A. muciniphila* was prepared in a dilution series to create a standard curve. The absolute copy number of the 16S rDNA gene of each sample was then calculated.

### *A. muciniphila* culture and oral supplementation

*A. muciniphila* culture and oral supplementation were performed as previously described^22^ with a few modifications. *A. muciniphila* (ATCC BAA-835) was grown in fresh pre-reduced Brain Heart Infusion (BHI) broth (Sigma-Aldrich, BD 237,500, USA) under anaerobic conditions (75% N_2_, 20% CO_2_, and 5% H_2_) at 37°C and incubated for 3–4 days. Afterward, cultures were centrifuged and condensed in anaerobic PBS containing 20% (vol/vol) glycerol to a concentration of ~ 1 × 10^10^ c.f.u/mL under strictly anaerobic conditions and stored at −80°C until use. The bacteria were incubated in a pre-reduced Tryptic Soy Agar medium (Sigma-Aldrich, BD 236,950, USA) for 4–5 days to determine the *A. muciniphila* counts (c.f.u./mL). *A. muciniphila* glycerol stocks were diluted with anaerobic PBS to a final concentration of 2 × 10^8^ viable c.f.u. per 0.2 mL. Mice were treated by oral gavage with 200 μL of either *A. muciniphila* suspension or anaerobic PBS three days a week (on days 1^st^, 3^rd^ and 5^th^) for four weeks, starting 48 h after the last gavage of antibiotics.

### SCFAs treatment

SCFA mix (67.5 mM acetate, 40 mM Butyrate) (Sigma-Aldrich, China) was added to drinking water as described previously.^25^ Mice were treated with drinking water with/without SCFAs for 4 weeks before SD until the behavior tests were completed.

### Microglia culture and SCFAs treatment

Primary microglial cultures were prepared from the cortical tissue of mouse pups on postnatal day 3 as previously described.^26^ Microglia were plated at a density of 2 × 10^4^ cells/cm^2^ and maintained in Dulbecco’s modified Eagle medium (DMEM) supplemented with 10% fetal bovine serum, penicillin (100 IU/mL), and streptomycin (100 μg/mL). SCFAs treatment was performed as previously described with a few modifications.^27^ SCFAs mixtures containing sodium acetate (Sigma-Aldrich, S2889, China) (236 μmol/L) and sodium butyrate (117 μmol/L) (Sigma-Aldrich, 303410, China) or vehicle (V) solution (H_2_O) were added to the cultured microglia for 15 min. The cells were then stimulated with LPS (100 ng/mL) for 24 h before all cultures were fixed for immunostaining.

### SCFAs treatment of neuron and neuron/microglia co-cultures

For mouse cortical neuron cultures, cortices from embryonic days (e) 14–17 embryos were dissected in a cold dissection buffer (1×HBSS).^28^ The meninges, blood vessels and choroid plexus were carefully separated; the tissue was finely minced into approximately 1-mm^3^ pieces with iris scissors for digestion. The suspension cells were obtained according to the manufacturer’s protocol.^28^ The cells were plated on poly l-lysine/laminin-coated coverslips at a density of 2.4 × 10^4^ cells/cm^2^ and cultured for 7 days *in vitro*. At DIV 7, microglia were plated onto primary neurons in a 1:3 microglia-to-neuron ratio. As described above, the SCFA mixture (sodium acetate 236 μmol/L and sodium butyrate 117 μmol/L) was added to the co-culture system or neuron cultures for 15 min, and LPS (100 ng/mL) was added for 24 h before all cultures were fixed using 4% paraformaldehyde (PFA) for immunostaining.

### Western blot assay

Western blotting was performed as previously described.^29^ Protein samples were extracted from hippocampal tissue in RIPA buffer supplemented with a protease and phosphatase inhibitor cocktail. After assessing the protein concentration with a BCA Protein Assay kit (Thermo Fisher Scientific, 23227, USA), they were degenerated by heating at 95°C for 10 min with loading buffer before cooling to room temperature and loaded onto 10% SDS-PAGE separation gels. The protein bands were then transferred to polyvinylidene fluoride (PVDF) membranes. Appropriate primary antibodies and HRP-conjugated secondary antibodies were used to detect the proteins of interest (details are shown in Supplementary Table S1). Immunoreactive bands were visualized using a chemiluminescent substrate. β-actin was used as the internal standard. Antibody information can be found in Supplementary Table S1. Immunoblots were quantified using ImageJ analysis software (Version 2.0, NIH, USA).

### Immunofluorescent staining

Mice were deeply anesthetized using isoflurane and transcardially perfused with 20–30 mL of ice-cold normal saline, followed by 15–20 mL of 4% PFA. The brains were removed and post-fixed in 4% PFA at 4°C for 6–8 h. After dehydration in 30% sucrose in 0.1 M Phosphate buffer at 4°C for 3–4 days, 30-µm thick coronal brain sections were obtained using a cryostat (Thermo, CryoStar N×50OPD). Free-floating sections were blocked with 5% donkey serum/0.3% Triton X-100 in PBS for 2 h at room temperature (RT), followed by incubation with primary antibodies (antibody information is shown in Supplementary Table S1) diluted at 4°C overnight. The sections were rinsed three times in PBS the morning before incubation with the corresponding secondary antibodies for 2 h at RT.

### Imaging and analyses

Immunolabeled proteins were imaged using a Nikon A1 confocal microscope (Nikon, Japan). The images for dentate gyrus synaptic marker analysis were captured using a 63× oil immersion objective lens and 3× zooms with an interval of 5 μm along the Z-axis. Iba1 and CD68 were captured using a 40× objective lens. Iba1 and synaptophysin (SYP) were captured using a 63× objective. VGLUT1 and PSD95 of primary neurons were captured using 60× objectives and 4× zooms using a Leica TCS SP8 STED 3X confocal microscope (Leica, Germany). Images were acquired from three sections representing the rostral, middle, and caudal regions of the hippocampal dentate gyrus. For imaging, we used three brain slices per mouse from three mice per experimental group, and three images were taken for each slice at random locations in the dentate gyrus, as defined by DAPI staining. For *in vitro* experiment, images from five fields per coverslip distributed in each quadrant or center with three replicates were taken for analysis. The acquired Z-stacks were then individually projected onto a two- dimensional plane. For the synaptic marker count per unit, the area was analyzed using the ImageJ program with a threshold of normalized fluorescence intensity. For synapses inside microglia, synaptic puncta labeled with SYP and Iba1 were imaged, as described above. Subtracted background and smooth images were obtained using ImageJ software. Microglia engulfment was quantitatively measured using the Imaris software 9.0.1. All experimental analyses were performed by a researcher who was blinded to the treatment groups.

### Metabolome analyses

#### Untargeted LC-MS/MS metabolomics

Approximately 500 µL blood samples were collected from the four groups of mice (Con/V, AKK/V, SD/V, and AKK/SD) after anesthetizing the mice with isoflurane. For serum extraction, blood samples were centrifuged at 3000×g for 10 min at 4°C and stored at −80°C. Metabolites were extracted from mouse serum using the MLPEx method.^30^ Briefly, 40 μL of serum was extracted using 160 μL of methanol/acetonitrile (1:1, v/v). After vortexing for 2 min, the mixture was incubated at −20°C for 1 h to precipitate proteins and centrifuged at 13,000 rpm for 15 min. The supernatant was collected and dried using a nitrogen concentrator. The dried residue was reconstituted in 100 µL of 50% acetonitrile before analysis by liquid chromatography-tandem mass spectrometry (LC-MS/MS), as reported previously.^31^

Untargeted metabolites data analysis was performed by dedicated Compound Discoverer 3.2 software. The Principal Component Analysis view was used to display the results of the principal component analysis. The principal component analysis reduces the dimensionality of the data set to a set of principal components, PC1 and PC2, where PC1 is the principal component with the most variance. Welch *t*-test was used for the comparison of Con/v vs. SD/V, SD/AKK vs. SD/V, and SD/AKK vs. Con/V. For the significant difference analysis, the *P*-value was set to 0.05 and the Log2 Fold Change parameter was set to 1. The volcano plot is a plot of the *P*-value, the result of a significance test, on the y-axis versus the Log2 fold change between two sample groups on the x-axis. The y-axis scale is the – Log10 of the *P*-value.

#### Targeted metabolomics

Blood sample preparation was performed as described in the previous Section. Serum samples (50 µL in 50% methanol) were dried in a speed vac to remove methanol before drying was performed in a lyophilizer. All the samples were re-dissolved in 100 µL of 0.1% formic acid. Then, these were analyzed by liquid chromatography-mass spectrometry (LC-MS/MS) as reported previously.^32^ The Peak area of each isotope-labeled internal standard was used to normalize that of the 7 SCFAs (Acetic acid, propanoic acid, butyric acid, isobutyric acid, valeric acid, isovaleric acid and hexanoic acid) with the same number of carbons.

### Bioinformatic analysis

The microbiomes were analyzed at the Illumina sequencing platform by the Beijing Genomics Institute (Beijing, China). High-quality reads for bioinformatics analysis were selected, and all of the valid reads from all samples were clustered into operational taxonomic units (OTUs) based on 97% sequence similarity using the Usearch (v7.0.1090_i86linux32). Then, OTUs were annotated by comparing representative sequences to the Greengene (V201305) and Ribosomal Database Project (RDP) (Release16, 20160930) using the RDP classifier (1.9.1). The difference comparisons among different groups were displayed by the principal coordinate analysis (PCoA) based on the UniFrac distance matrix. Alpha-diversity (Shannon index and Simpson index) indices were calculated by Wilcoxon Rank-Sum Test. Beta-diversity analysis was performed using weighted UniFrac distances. LEfSe (Linear discriminant analysis effect size) analysis was performed under the following conditions: the *P*-value for the factorial Wilcoxon tests among classes was 0.05 and the threshold on the logarithmic LDA score for discriminative features was 2.0 (the code from https://github.com/SegataLab/lefse). The analysis was conducted using R (V3.5.1).

### Statistical analysis

Results are presented as the mean ± SEM, and differences were considered significant when *P* < .05. Statistical analyses were performed using Prism 7.0 (GraphPad, USA). Student’s *t*-test was used to compare two groups with a normal distribution, while One-way ANOVA with Tukey’s multiple comparisons was used for comparisons of three or more groups. Correlations were analyzed using linear regression.

## Results

### Altered gut microbiome exacerbates cognitive impairment in SD mice

**Figure 1.**
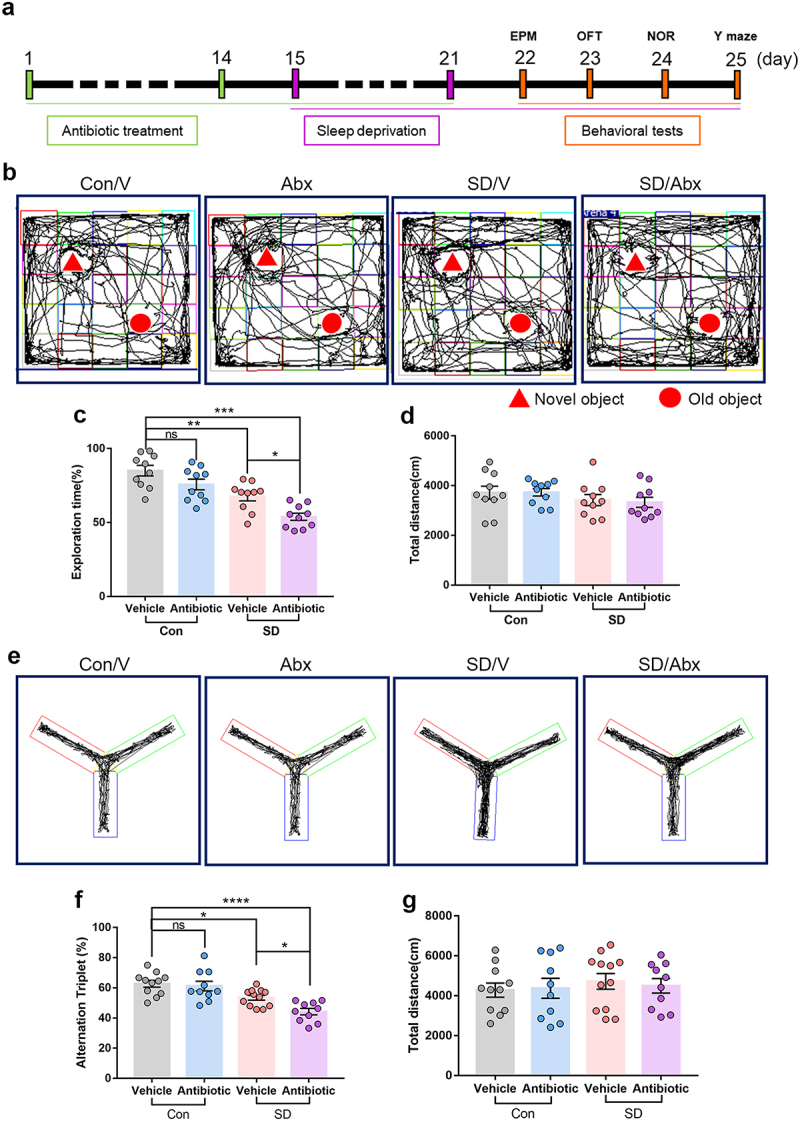
The effects of antibiotic pretreatment on the cognitive performance in SD mice.

To assess the role of the gut microbiome in SD, we tested the effect of antibiotic or vehicle pre-treatment on SD-induced cognitive impairment in mice (Figure 1a). First, the gut microbiome of mice was depleted using an antibiotic cocktail for 14 days (Supplementary Figure S3). SD was induced on day 15–21. Behavioral performance was quantified using the open-field, elevated plus maze, novel object recognition, and Y-maze tests after 7 days of SD. The open-field, elevated plus maze, novel object recognition, and Y-maze tests were conducted during the last cycle of SD between 9:00 and 16:00. The exploration time of SD mice decreased significantly compared to that of mice in the control groups (Figure 1b,c, Con/V 85.02 ± 3.58% vs. SD/V 67.70 ± 3.08%, *P* = .0027) and the spontaneous alteration index in the Y maze reduced in SD mice as well (Figure 1e,f, Con/V 62.69 ± 2.26% vs. SD/V 53.45 ± 1.61%, *P* = .0274). Compared with SD mice that received vehicle only, SD mice that received antibiotic treatment exhibited worse performance for the novel object recognition test (Figure 1b,c, SD/Ab 53.89 ± 2.44% vs. SD/V 67.70 ± 3.08%, *P* = .0210), and reduced spontaneous alternation index (Figure 1e,f, SD/Ab 44.24 ± 2.10% vs. SD/V, *P* = .0334). The total distance in the NOR and Y-maze tests was similar among the groups (Figures 1d and g). No differences were found in the OFT and EPM tests (Supplementary Figure S4). These data indicate that disrupting the gut microbiota before SD worsens cognitive impairment in SD mice.

(a) Schematic of antibiotic pretreatment and the mouse model for sleep deprivation (SD). (b) Representative tracks of mice in each group during the novel object recognition (NOR) test. (c) Percentage of exploration time in mice from different groups in NOR test. (d) Total distance traveled among different groups. *n* = 10 mice per group. (e) Representative track images of mice from different groups in the Y maze test. (f) Spontaneous alternation index of mice in all groups from the Y maze test. (g) Total distance mice traveled in the Y maze test. *n* = 10-12 mice per group. Abx, Antibiotic cocktail. (One-way ANOVA with Tukey’s multiple comparisons tests, *P < 0.05, **P < .01, ***P < .001, ****P < .0001, ns, no significant difference).

### Inoculation with microbiota from SD mice induces cognition impairment in healthy mice

**Figure 2.**
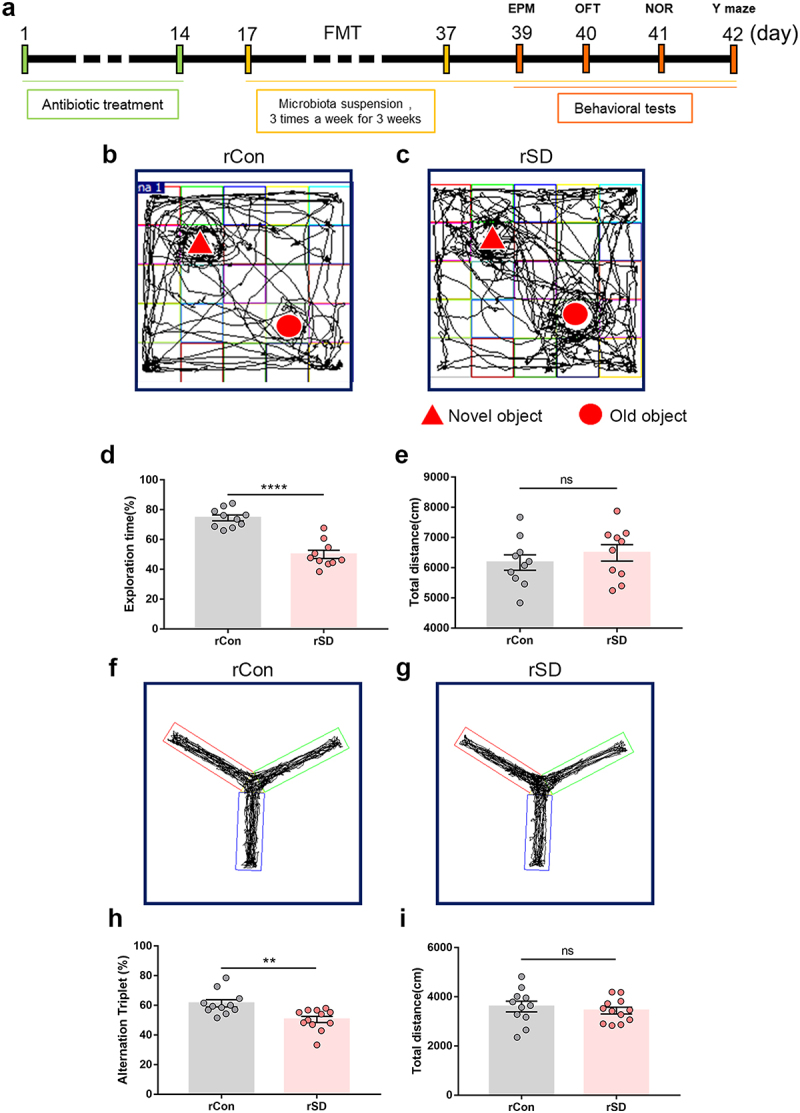
The effects of fecal microbiota transplant on cognitive performance in mice.

To examine whether the SD-associated gut microbiome is involved in cognitive impairment, fecal microbiota from sleep-deprived mice or normal sleep (control) mice were transplanted into 5-week old mice that were pretreated with the antibiotic cocktail. Cognitive function was evaluated 48 h after the last transplantation session (Figure 2a). Compared with the mice that received control mice microbiota (rCon), mice that received SD mice microbiota (rSD) exhibited a decreased preference for the novel object (Figure 2b–d, rSD 50.03 ± 2.78%, vs. rCon 74.51 ± 1.98%, *P* < .0001), and lower spontaneous alternation index (Figure 2f–h, rSD 50.47 ± 2.07%, vs. rCon 61.38 ± 2.40  %, *P* = .0024). The total distance traveled in the NOR and Y-maze fields was similar between the groups (Figures 2e and i). The performance in the OFT and EPM did not differ between the groups (Supplementary Figure S5), indicating no obvious change in anxiety behavior. These observations demonstrate that gut microbiota transplantation from SD mice induces cognitive impairment in the recipient mice.

(a) Schematic of fecal microbiota transplant (FMT) experiments. (b and c) Representative tracks of mice in different groups during NOR test. (d) Percentage of exploration time in mice from different groups in NOR test. (e) Total distance traveled among different groups. *n* = 10 mice per group. (f and g) Representative track images of mice from different groups in the Y maze test. (h) Spontaneous alternation index of mice in all groups from Y maze test. (i) Total distance traveled of mice in a Y maze test. *n* = 10-12 mice per group. (Student’s *t* test, **P < .01,***P < .001, ns, no significant difference). rCon, the mice received control mice microbiota. rSD, the mice received SD mice microbiota.

### SD induces dysbiosis of gut microbiota and reduction in *A.* *muciniphila* in mice

**Figure 3.**
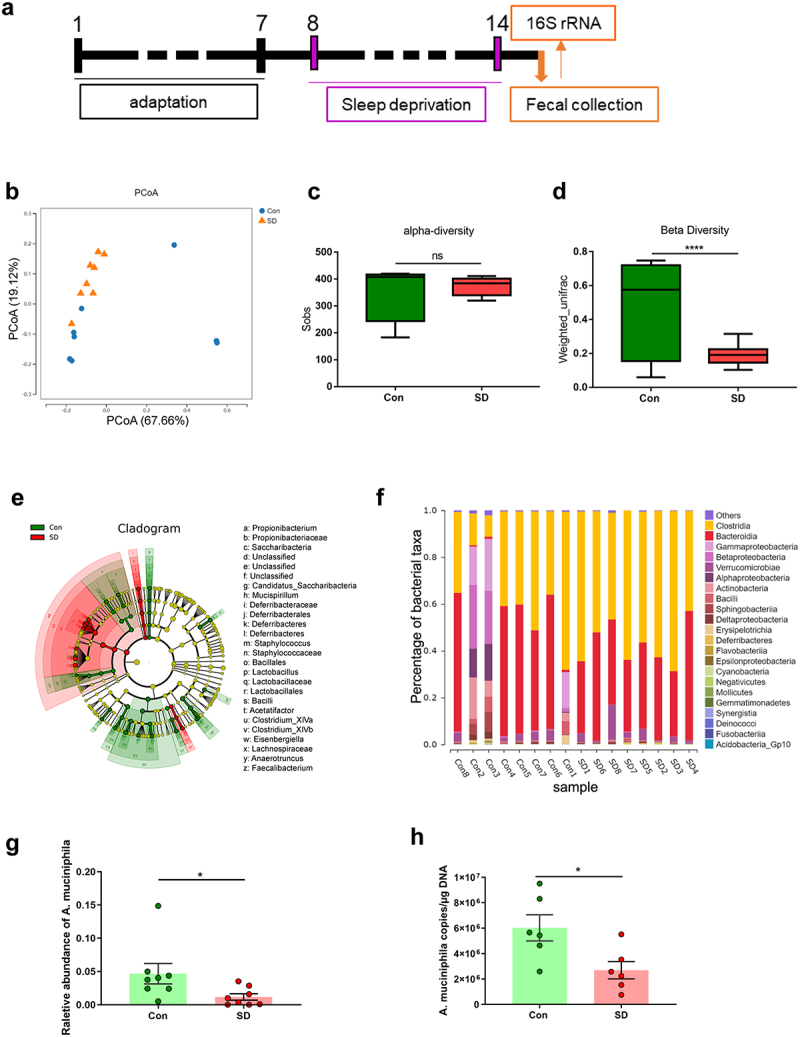
SD alters gut microbiome composition in mice.

To elucidate how the gut microbiota influences brain function in SD mice, we used 16S rDNA sequencing to assess the composition of the fecal microbiome of SD mice and controls (Figure 3a). Principal coordinate analysis (PCoA) showed that the pattern between SD and control mice was substantially different (Figure 3b and Supplementary Table S2), while no significant changes in the total number of OTUs (alpha diversity) were observed (Figure 3c, *P* = .5836). Meanwhile, we evaluated the beta diversity between groups according to the UniFrac distance metric, which revealed a significant reduction in beta diversity in the gut microbiota of SD mice compared to controls (Figure 3d, *P* < .0001). The taxonomic cladogram obtained from LEfSe analysis was shown in Figure 3e, and the LDA values of each group was shown in Supplementary Figure S6. Next, we calculated the percentage of bacterial taxa in each group (Figure 3f). *Bacilli, Betaproteobacteria* and *Sphingobacteria* were significantly decreased. However, *Clostridia* were markedly enriched in the SD group (Figure 3f). Consistent with previous reports,^33,34^ the relative abundance of *A. muciniphila* was lower in SD mice than in the controls (Figure 3g, *P* = .0481). Thereafter, changes in *A. muciniphila* were validated by qPCR. The results showed that *A. muciniphila* was significantly decreased in the cecal content of mice after 7 days of SD (Figure 3h, SD 2,692,656 ± 683019 copies/μg DNA vs. Con 6,021,399 ± 1025271, *P* = .0222). These data indicate that SD alters the gut microbiota and reduces *A. muciniphila* content.

(a) Schematic of SD experiments. (b) Principal coordinates analysis (PCoA) of weighted UniFrac distances from the averaged rarefied 16S rDNA gene dataset between control and SD mice. (c) The total number of observed OTUs (alpha diversity). (d) β diversity comparisons based on the weighted_unifrac distances. (e) Taxonomic cladogram obtained from LEfSe analysis. (f) Comparison of key species differences. (g) The relative abundance of *Akkermansia muciniphila* (*A. muciniphila*, AKK). *n* = 7-8 mice per group. (h) The concentration of *A. muciniphila* in cecal content was assessed by qPCR. *n* = 6 mice per group. (Student’s *t* test, *P < .05, ****P < .0001, ns, no significant difference).

### *A.* *muciniphila* colonization alleviates SD-induced cognitive impairment in mice

**Figure 4.**
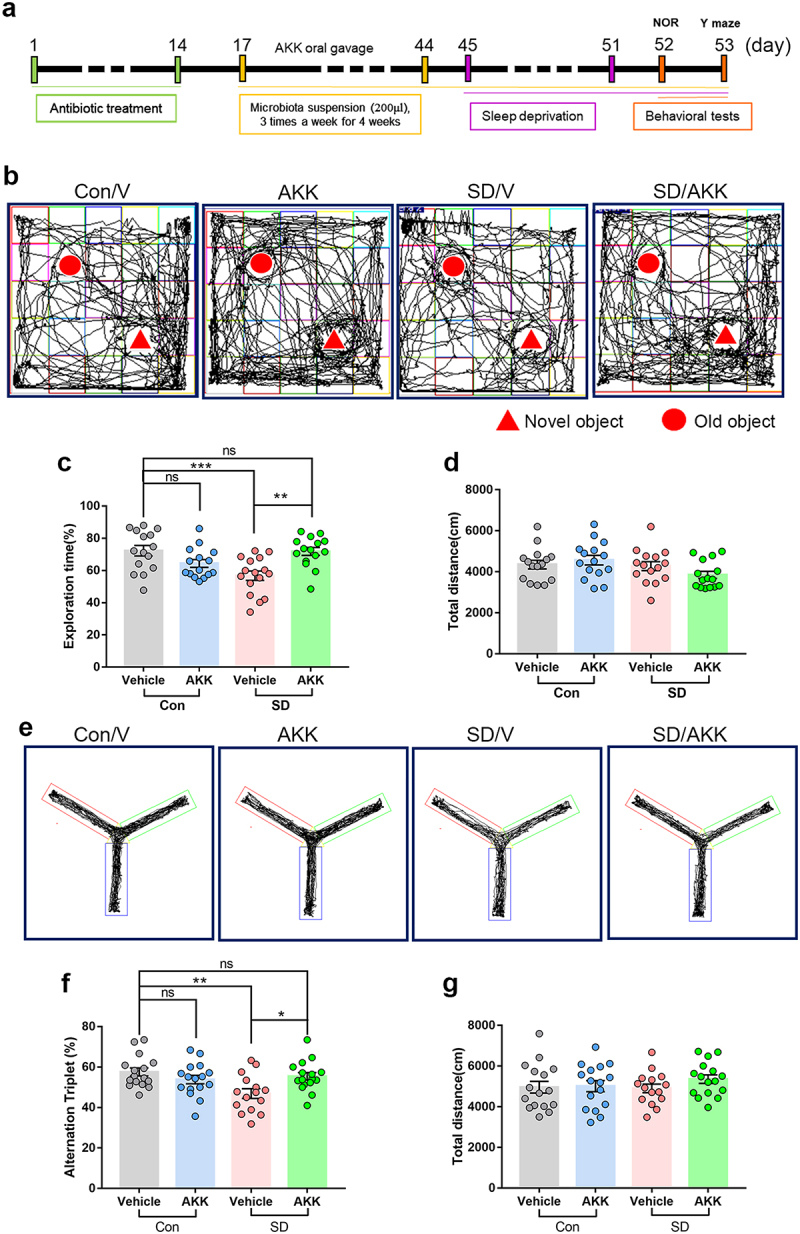
*A. muciniphila* supplement restores SD-induced cognitive deficits in mice.

To evaluate whether supplementation with *A. muciniphila* prevents cognitive impairment induced by SD, we supplemented *A. muciniphila* (ATCC BAA-835) or its storage buffer (anaerobic PBS) to C57BL/6 mice via oral gavage after antibiotic cocktail-based microbiota depletion (Figure 4a). qPCR was used to quantify the copy number of *A. muciniphila* in the cecal contents. The results showed that *A. muciniphila* colonization increased significantly after oral supplementation (Supplementary Figure S7). The NOR test showed that the reduction in exploration time for the novel object in SD mice was prevented by *A. muciniphila* pretreatment (Figure 4b,c, SD/AKK 71.83 ± 2.46%, vs. SD/V 56.79 ± 2.89%, *P* = .0012). The Y-maze results indicated that the spontaneous alternation index drop observed in SD mice was also blocked by *A. muciniphila* pretreatment (Figure 4e,f, SD/AKK 55.44 ± 1.88%, vs. SD/V 46.88 ± 2.39%, *P* = .0271). The total distance traveled in the NOR and Y-maze tests was similar between the groups (Figures 4d and g). These results highlight the efficacy of *A. muciniphila* supplementation as a possible precautionary treatment for SD-induced cognitive impairment in mice.

(a) Schematic of *A. muciniphila* oral administration experiments. (b) Representative tracks of mice in each group during the NOR test. (c) Percentage of exploration time of mice from different groups in NOR test. (d) Total distance traveled among different groups. *n* = 15-16 mice per group. (e) Representative track images of mice from different groups in the Y maze test. (f) Spontaneous alternation index of mice in all groups from Y maze test. (g) Total distance mice traveled in the Y maze test. *n* = 15-16 mice per group. (One-way ANOVA with Tukey’s multiple comparison tests, *P < .05, **P < .01, ***P < .001, ns, no significant difference).

### *A. muciniphila* colonization ameliorates SD-induced synapse loss in mice

**Figure 5.**
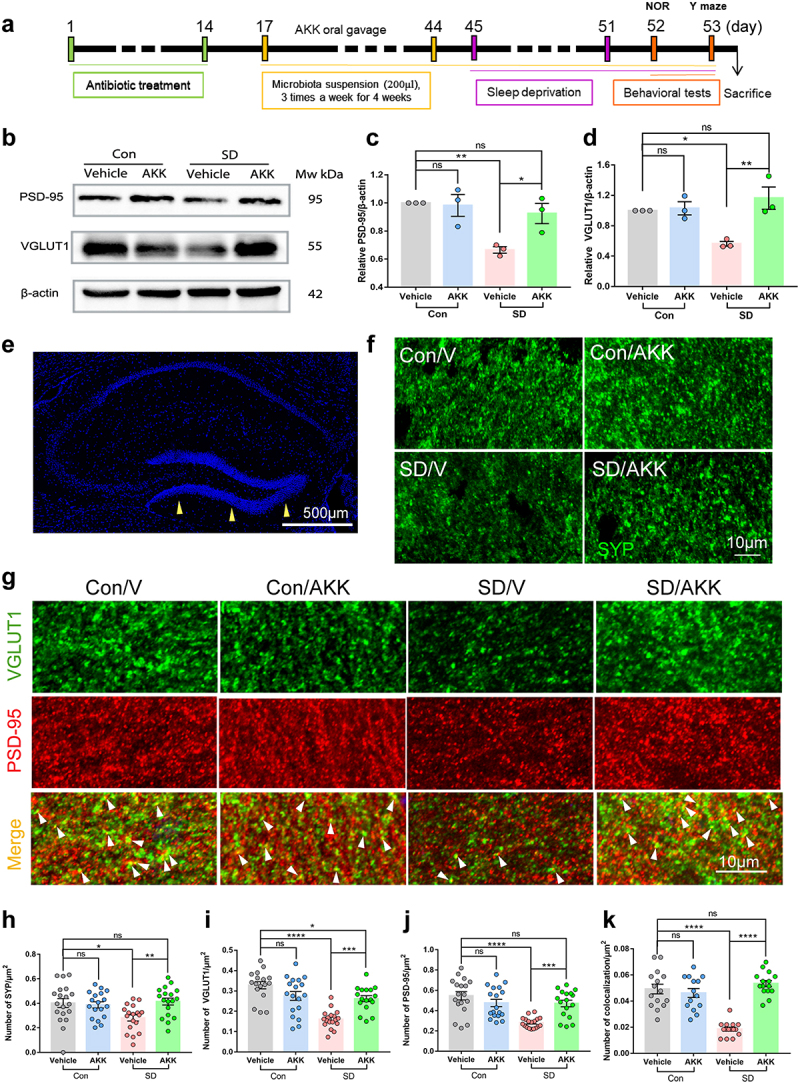
*A. muciniphila* supplement attenuates synaptic loss in SD mice.

To explore how *A. muciniphila* supplementation attenuated SD-induced cognitive impairment, the expression of synaptic proteins VGLUT1, a marker of excitatory presynaptic elements, and PSD-95, a universal postsynaptic marker, in the hippocampus was assessed using western blotting (Figure 5a). The results showed that VGLUT1 and PSD-95 protein levels were significantly reduced in SD mice that were administered vehicle only compared with the control group. However, *A. muciniphila* administration prevented synaptic loss in the SD mice (Figure 5b–d). Next, we detected the immuno-density of SYP, VGLUT1, and PSD-95 in the dentate gyrus by immunofluorescence staining (Figure 5e–g). The density of SYP, VGLUT1, PSD-95, and VGLUT1/PSD-95 co-localization was significantly decreased in the SD/V group, while *A. muciniphila* treatment blocked these changes (Figure 5h–k). Collectively, these findings indicate that AKK supplementation may preserve synaptic loss in the dentate gyrus of SD mice.

(a) Schematic of *A. muciniphila* oral administration experiments. (b, c) Effects of *A. muciniphila* treatment on VGLUT1 protein levels in the hippocampus of each group. (b, d) PSD-95 protein levels in the hippocampus of each group. *n* = 3 mice per group. (e) The yellow arrowheads in the DAPI figure denote the imaged locations of the magnified synaptic markers below. Scale bar = 500 μm. The representative images of SYP (f), VGLUT1 and PSD-95 (g) in the dentate gyrus of each group. Scale bar = 10 μm. (h) Quantification of SYP labeling. (i) Quantification of VGLUT1 labeling. (j) Quantification of PSD-95 labeling. (k) Quantification of VGLUT1 and PSD-95 double-labeled puncta (white arrowheads in g). *n* = 15-18 areas per group. (One-way ANOVA with Tukey’s multiple comparison tests, *P < .05, **P < .01, ***P < .001, ****P < .0001, ns, no significant difference).

### *A.* *muciniphila* reduces extensive microglia activation and synapse engulfment in the hippocampus of SD mice

**Figure 6.**
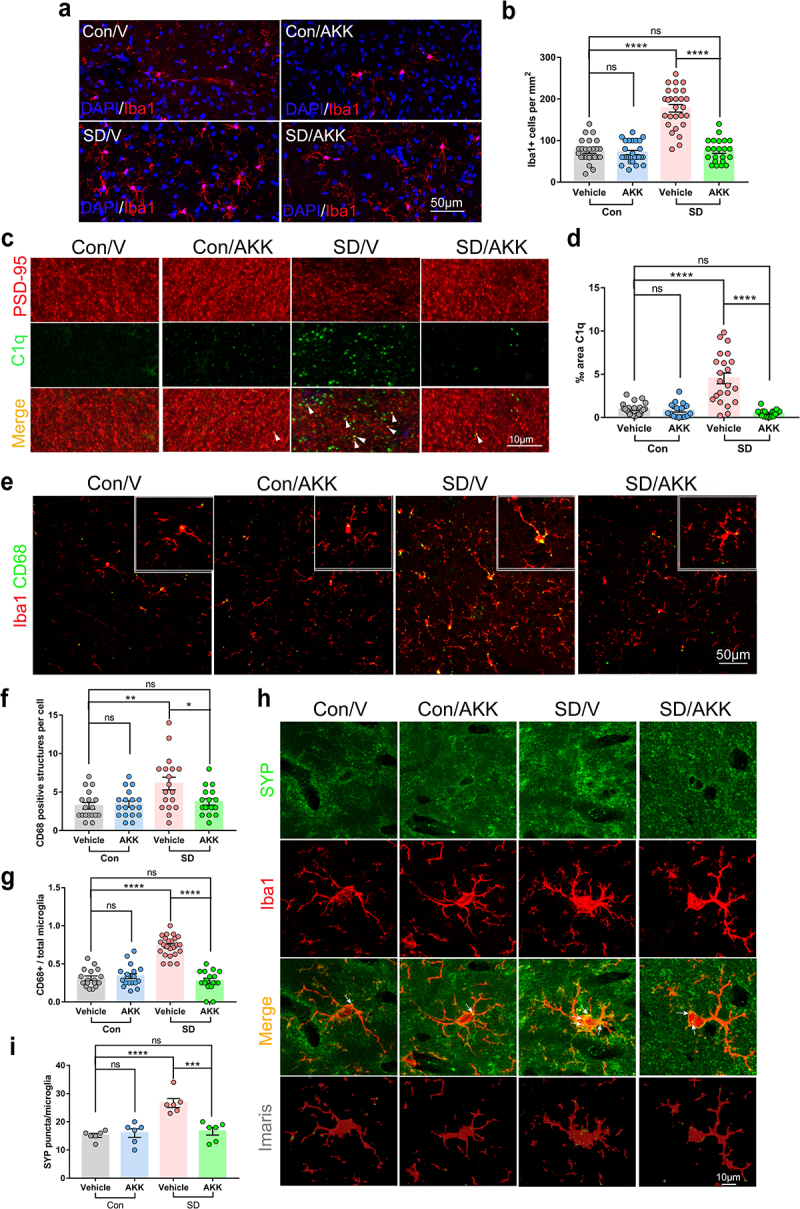
*A. muciniphila* supplement inhibited extensive microglial activation and synaptic engulfment in the hippocampus of SD mice.

To assess whether 7 days SD activated microglia in the mouse hippocampus and whether *A. muciniphila* pretreatment modulated its activation, we conducted immunofluorescent staining for Iba1, a recognized marker for microglia, and quantified microglia density in the hippocampus of all groups (Figure 6a). AKK supplementation alone did not alter microglial activation. However, SD significantly increased Iba1-positive staining in the mouse hippocampus, whereas AKK pretreatment significantly reduced microglial activation in SD mice (Figure 6a,b). As the complement protein C1q is a key protein for microglia pruning and synapse engulfment,^35^ we assessed C1q expression by immunostaining. The results showed that C1q immunoreactivity was elevated in SD/V mice compared with that in controls. AKK treatment significantly reduced C1q expression in the hippocampi of mice after SD (Figure 6c,d). Moreover, CD68, a lysosomal marker, was used to evaluate the phagocytic activity of microglia in the mouse hippocampus. CD68-positive lysosomes accumulated in the microglia of SD/V mice as compared to controls, but AKK pretreatment significantly decreased CD68-positive staining in the microglia of mice after SD (Figure 6e–g). Finally, an increased number of SYP puncta was found in the Iba1+ microglia of SD/V mice. However, AKK pre-treatment blocked this change (Figure 6h,i). These results indicate that *A. muciniphila* administration inhibited microglial overactivation and synaptic engulfment in the hippocampus of mice after SD.

(a) Representative immunofluorescence images of microglia (Iba1+) in the hippocampus of Con/V, Con/AKK, SD/V and SD/AKK mice. Scale bar = 50 μm. *n* = 22-27 areas per group. (b) The density of Iba1+ cells in the hippocampus of each group.(c) Representative images of C1q (Green) immunoreactivity in the hippocampus of each group and (d) quantification. Scale bar = 10 μm. (e, f and g) Iba1 and CD68 co-stain in the hippocampus of each group. Scale bar = 50 μm. *n* = 17-18 areas per group. (h and i) Confocal and Imaris reconstruction images showing SYP puncta within Iba1+ microglia in the hippocampus in each group (white arrows). *n* = 6 cells per group. (One-way ANOVA with Tukey’s multiple comparison tests, *P < .05, **P < .01, ***P < .001, ****P < .0001, ns, no significant difference).

### *A.* *muciniphila* colonization reversed serum SCFAs reduction in SD mice

**Figure 7.**
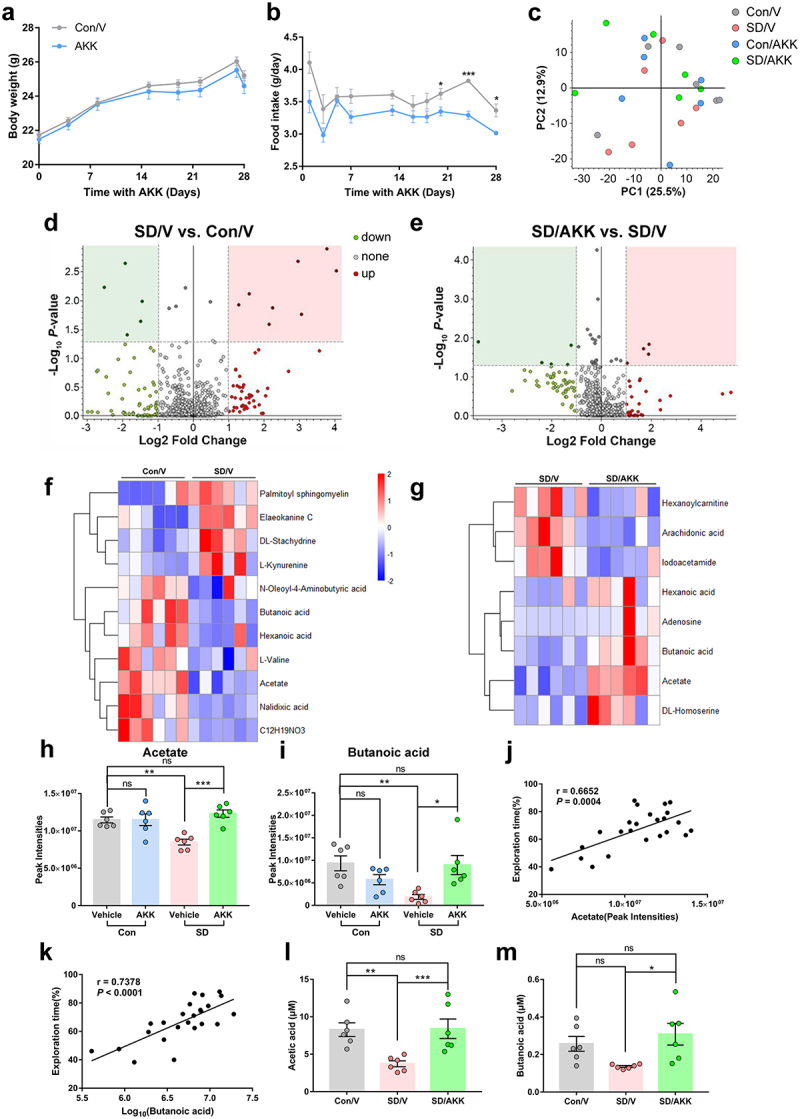
The effects of *A. muciniphila* colonization on metabolic dysfunction-associated SD in mice.

Body weight was similar among the groups throughout the 4 weeks of AKK treatment period (Figure 7a). Food intake was lower in AKK-supplemented mice than that in vehicle controls from the third week of AKK treatment (Figure 7b). Since gut microorganisms cannot translocate to the central nervous system, we proposed that specific microbiome-regulated metabolites from the gut microbiota that can cross the blood-brain barrier may affect hippocampal neurons in SD mice. Thus, we used untargeted metabolomic analysis to identify gut microbiome-associated metabolites that were differentially abundant in the serum of AKK- and vehicle-supplemented mice after SD. Principal coordinate analysis (PCA) revealed variance scores for PC1 of 25.5% and PC2 of 12.9%. (Figure 7c). Compared to the Con/V group, the SD/V group displayed five downregulated and eight upregulated metabolites (Figure 7d). Five metabolites were decreased and four were increased in the SD/AKK group compared to the SD/V group (Figure 7e). Compared to the Con/V group, the SD/AKK group displayed seven downregulated and fourteen upregulated metabolites (Supplementary Figure S8). The enrichment of representative metabolites in serum in the SD/V and SD/AKK groups is shown in (Figures 7f,g). Specifically, acetate and butanoic acid were significantly decreased in the SD/V mice, and AKK pretreatment prevented this change (Figure 7h,i).

(a) Body weight and (b) food intake of control and AKK supplementary mice. *n* = 12 mice per group. (Two-tailed*t*-test) (c) Principal coordinates analysis (PCoA) of metabolite profiles in serum samples. (d and e) Differentiation of metabolites between the SD/V and control mice (d), and between the SD/AKK and SD mice revealed by volcano plot (e). The enrichment of representative metabolite in serum upon SD/V (f) and SD/AKK group (g). (h) Acetate and (i) butanoic acid relative levels in the serum of mice. (j and k) The exploration percentage positively correlates with gut metabolites acetate (j) and butanoic acid (k). (l and m) The concentrations of acetate and butanoic acid in the serum of mice. *n* = 6 mice per group. (One-way ANOVA with Tukey’s multiple comparison tests, *P < .05, **P < .01, ***P < .001, ns, no significant difference).

Furthermore, to better understand the connection between SCFAs levels and cognitive function, a correlation analysis was performed between the levels of several SCFAs and percentage of exploration time. The results demonstrated that the serum levels of acetate and butanoic acid were positively correlated with the percentage of exploration time (Figure 7j,k). Additionally, the results of SCFA-targeted metabolomics showed that the serum concentrations of acetate and butanoic acid were decreased in SD mice compared with those in control mice, which were significantly restored by oral administration of AKK (Figure 7l,m). We then tested the effects of SCFAs (acetate and butanoic acid) pre-supplements in SD mice and conducted behavioral analysis (Supplementary Figure S9a). The NOR test showed that the reduction in exploration time for the novel object in SD mice was prevented by SCFAs pretreatment (Supplementary Figure S9b, SD/SCFAs 69.16 ± 3.658%, vs. SD/V 55.81 ± 3.70%, *P* = .0178). The Y-maze results indicated that the spontaneous alternation index drop observed in SD mice was also blocked by SCFAs pretreatment. (Supplementary Figure S9d, SD/SCFAs 56.87 ± 2.97%, vs. SD/V 46.19 ± 2.03%, *P* = .0165), while the total distance traveled in the NOR and Y-maze tests was similar among groups (Supplementary Figure S9c and e). The above results indicate that SCFAs pretreatment alleviated SD-induced cognitive impairment. These results suggest that AKK treatment restored acetate and butanoic acid levels, which may be the basis for its protective effect against SD-induced cognitive dysfunction.

### SCFAs prevent synapse loss via reducing synapse engulfment of microglia

**Figure 8.**
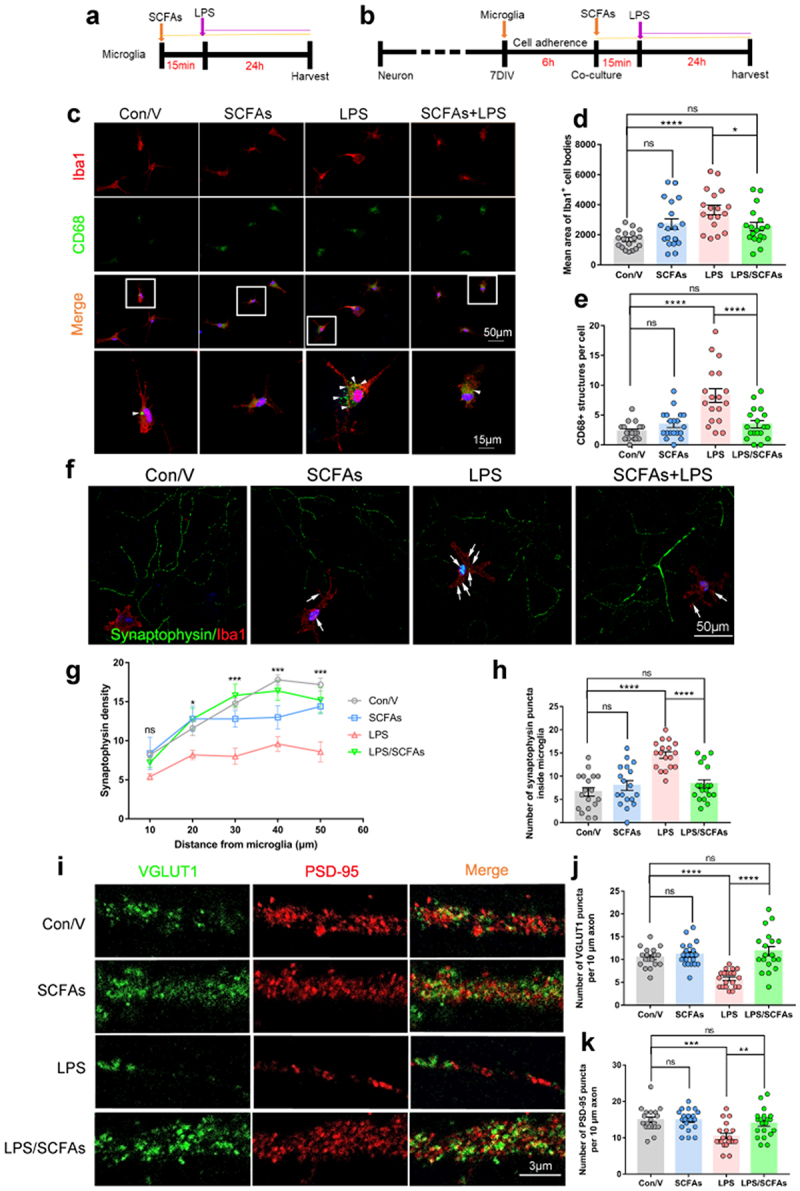
Short-chain fatty acids (SCFAs) alleviated synapse loss by inhibiting synaptic engulfment of microglia *in vitro*.

To verify that SCFAs restoration mediates the reduced microglial activation and synaptic engulfment, we conducted double-immunofluorescent staining for Iba1/CD68 and Iba1/SYP in the hippocampus of mice. The data showed that SCFAs pretreatment significantly reduced the Iba1-positive staining in the hippocampus of SD mice (Supplementary Figure S10a and b). Additionally, SCFAs pretreatment also resulted in a reduction of CD68-positive dots within the Iba1+ microglia (Supplementary Figure S10a, c and d). Meanwhile, the number of SYP-positive puncta in the Iba1+ microglia of SD/V mice was also decreased in SCFAs pretreated mice as compared to SD/V mice (Supplementary SFigure 10e and f). Furthermore, we employed an LPS stimulation model of cultured primary microglia to mimic microglial activation after SD (Figure 8a). Microglia stimulated with LPS exhibited larger areas of Iba1-positive cell bodies compared to Con/V, while SCFAs inhibited this change induced by LPS stimulation. (Figure 8c,d). Additionally, it was found that the accumulation of CD68-positive lysosomes was enhanced in microglia treated with LPS compared with the control ones, but it appeared to decrease in the microglia treated with LPS plus SCFAs (Figures 8c–e). These results indicated that SCFAs may inhibit the phagocytic activity of microglia during LPS-induced inflammation.

(a) SCFAs treatment of primary microglia. (b) SCFAs treatment of neuron/microglia co-culture. (c) Representative images of Iba1 (red), CD68 (green) and DAPI (blue) in the primary microglia from each group. Scale bars, 50 µm (overview) and 10 µm (enlarged). (d) Quantification of mean areas of Iba1-positive cell bodies and (e) CD68-positive lysosomes in microglia. (f) Representative images of Iba1 (red), synaptophysin (SYP) (green) and DAPI (blue) in the primary neuron/microglia co-cultures. (g) Quantification of synaptophysin density. (h) The number of synaptophysin puncta inside microglia. (i) Representative images of VGLUT1 (green), PSD-95 (red) of primary neurons in the primary neuron/microglia co-cultures. (j and k) Quantification of VGLUT1 and PSD-95 densities. *n* = 18-20 fields per group. (One-way ANOVA with Tukey’s multiple comparisons, *P < .05, **P < .01, ***P <.001, ****P <.0001, ns, no significant difference).

VGLUT1 and PSD-95 protein levels were significantly increased in SD mice pretreated with SCFAs compared with the SD/V group (Supplementary Figure S10g-i). Therefore, to investigate whether SCFAs prevent synapse loss via reducing synapse engulfment of microglia. We used a co-culture system in which microglia were added to primary neurons cultured for 7 days *in vitro*. SCFAs were pre-administered to the co-culture system 15 min before LPS treatment. After 24 h, cells were fixed for immunostaining (Figure 8b). Quantitative analyses of neuron-microglia co-cultures revealed decreased synaptophysin density in neuron/microglia treated with LPS compared to that in control neurons (Figure 8f,g). Moreover, a significant increase in synaptophysin-positive puncta inside microglia was found in co-cultures treated with LPS compared to the control cells. However, pretreatment with SCFAs blocked this change in co-culture (Figures 8f–h). In primary cultured neurons, LPS treatment did not alter VGLUT1 or PSD95 expression (Supplementary Figure S11), indicating that other necessary factors outside the neurons are responsible for synaptic loss. In contrast, VGLUT1 and PSD95 were significantly reduced in neuron/microglia co-cultured with LPS compared to control neurons. SCFAs administration significantly rescued VGLUT1 and PSD95 expression in the neuron/microglia co-culture after LPS stimulation (Figures 8i and k). Together, these results suggest that SCFAs protect against synapse loss by inhibiting synaptic engulfment of microglia.

## Discussion

In this study, we demonstrated that alterations in gut microbiota were associated with SD-induced cognitive impairment. Transplanting the “SD-derived microbiota” into specific pathogen-free mice impaired the cognitive function of the recipient mice. We analyzed the gut microbiome and found a significant reduction in *A. muciniphila* in SD mice. Notably, the administration of *A. muciniphila* alleviated cognitive dysfunction and prevented synaptic reduction in the hippocampi of SD mice. The extensive microglial activation and synapse engulfment observed in the hippocampus of SD mice can be inhibited by *A. muciniphila* pre-treatment. Moreover, *A. muciniphila* pretreatment elevated the serum levels of acetate and butanoic acid in SD mice. The levels of acetate and butanoic acid in the serum were positively correlated with the performance of mice in the NOR test. SCFAs pretreatment improved SD-induced cognitive impairment in mice and reduced microglial activation and synaptic engulfment *in vivo*. Furthermore, SCFAs may inhibit synaptic loss by reducing synapse engulfment of microglia *in vitro*. Therefore, our results suggest a potential modulatory role of the gut microbiome in SD-induced cognitive impairment and reveal that *A. muciniphila* supplementation or SCFAs delivery may be a novel therapeutic strategy for preventing SD-induced cognitive dysfunction.

It was reported that brain dysfunction was accompanied by alterations in the gut microbiota in rats that experienced a 7-day paradoxical SD.^33^ There was also a possible correlation among sleep quality, the composition of the gut microbiome, and cognitive flexibility in healthy older adults.^13^ However, the causal relationship between gut dysbiosis and brain dysfunction in sleep-deprived subjects is still missing. Our results showed that antibiotic perturbation of gut microbiota worsened spatial and object memory in SD mice with no significant change in anxiety levels, which indicated that a few protective microbes remained in the gut of SD mice, which could be erased by antibiotic pretreatment. In addition, we confirmed the causal relationship between gut microbiota alteration and cognitive impairment by transferring “SD-derived microbiota” to recipient mice, which induced a decline in cognitive function. This was following a previous study that indicated that germ-free (GF) mice that received FMT from human participants after 48-h SD exhibited impaired cognitive behavior.^14^ The same study also revealed that sleep deprivation in GF mice exhibited less cognitive disturbance than control mice, indicating a possibility that the “bad microbiota,” which may cause or worsen the cognitive impairment, was increased after SD in non-GF mice. These results highlight the importance of deciphering microbiota alterations in the gut following SD.

Previous studies have shown that the abundance of *A. muciniphila* was distinctly reduced after SD in rats who experienced 7 days chronic paradoxical SD.^33^ Mice subjected to 3 days SD also exhibited downregulation in commensal bacteria, including *Akkermansia*, *Bacteroides* and *Faecalibacterium*, and upregulation in the pathogen *Aeromonas*.^34^ Moreover, it was reported that *A. muciniphila* improves cognitive function in high-fat diet-induced obesity mice^36^ and a rat model of Nonalcoholic steatohepatitis (NASH).^37^ In the current study, *A. muciniphila* supplementation inhibited the development of cognitive impairment after SD, suggesting that *A. muciniphila* may be a probiotic that may prevent SD-induced cognitive dysfunction.

Emerging studies suggest that the interaction of microglia with synapses contributes to synaptic remodeling during neural development, sleep and memory formation.^38–40^ SD could promote extensive microglia activation.^18^ Inhibiting microglial activation could reduce spatial memory decline in rats that underwent 48-h SD.^19^ It was also shown that SD induced extensive microglia activation and promoted phagocytic activity.^18^ We speculated that the status of microglia may be involved in preserved cognitive function provided by *A. muciniphila* in SD mice. Indeed, our data are consistent with these results, showing increased microglial activation in the hippocampi of SD mice. Further exploration showed that *A. muciniphila* pre-supplementation significantly inhibited microglial activation and reduced CD68/Iba1, C1q/Iba-1, and SYP/Iba-1 co-immunofluorescence staining. These results indicated that microglial phagocytosis of synapses was blocked by *A. muciniphila* pretreatment in SD mice.

However, exactly how does *A. muciniphila* pre-supplementation inhibit microglial activation and synaptic loss? We analyzed the gut metabolite changes induced by SD, and further compared the metabolites of mice that suffered from SD with or without AKK supplementation. The analysis showed that acetate and butanoic acid levels were significantly decreased in the SD/V group compared to those in the controls. However, these changes were inhibited by AKK supplementation. Consistent with our results, one study displayed that SCFAs levels were decreased in fecal samples from humans who experienced 24 h or 48 h SD, and in the fecal samples and serum of mice that received SD human-derived fecal microbiota transplantation.^14^ The study on the growth, metabolism, and morphology of *A. muciniphila in vitro* has shown that the main metabolites produced were SCFAs (acetic acid and butyric acid) under dynamic culture using porcine mucin or human mucin.^41^ In our study, the administration of AKK recovered the dropped SCFAs levels in the serum of SD mice. Meanwhile, acetate and butanoic acid levels in gut metabolites were significantly positively correlated with the percentage of exploration time in the NOR test. SCFAs pretreatment alleviated cognitive impairment in sleep-deprived mice. These results suggest that the effect of AKK pre-treatment on cognitive impairment is associated with SCFAs production in mice after SD.

Acetate and butanoic acid belong to SCFAs that have been shown to readily cross the blood-brain barrier^42,43^ and affect brain function in development, health, and disease.^44^ SCFAs may modulate the growth, survival and differentiation of neurons and synapses in the CNS by altering the levels of neurotransmitters and neurotrophic factors.^45–47^ In addition, SCFAs may affect the microglia maturation in neurodevelopmental and neurodegenerative disorders.^25,48^ Multiple literatures pointed out that sodium butyrate decreased microglia activation and inhibited pro-inflammatory cytokines secretion in pathologic conditions.^49–51^ In consistence with the previous research,^18^ our results showed that chronic SD activates microglia and promotes their phagocytic activity toward synaptic elements. AKK pretreatment inhibited the decrease in serum SCFAs levels in SD mice. Microglial activation and synapse loss were also alleviated by SCFAs pretreatment in the hippocampi of SD mice. To further corroborate the causal relationship between SCFAs levels and microglial phagocytic activation, we tested the effects of acetate and butanoic acid on LPS-induced microglial phagocytosis of neuronal synapses in a microglia-neuron co-culture system. Microglial phagocytosis was activated by exposure to LPS *in vitro* as previous reports.^52^ SCFAs treatment inhibited microglia engulfment and significantly reduced synaptic loss. Thus, SCFAs produced by AKK pretreatment may be the key components that translocate to the CNS and inhibit microglial activation, thus preventing synaptic loss and preserving cognitive function after SD.

*A. muciniphila* is a second-generation probiotic that has been tested in humans with insulin resistance. It is worth exploring whether AKK supplementation provides cognitive benefits to patients with sleep disorders. Other gut metabolite changes in SD mice were rescued by AKK supplementation, as shown in Figure 7d–g. Exploration of the specific effects of these metabolites will provide a better understanding and strategy for the composition of potential nutritional supplements for patients with sleep disorders.

## Conclusion

In conclusion, we identified that dysbiosis of the gut microbiota contributes to hippocampal synapse loss in SD mice with cognitive impairment. More importantly, *A. muciniphila* supplementation alleviates cognitive dysfunction and prevents synaptic phagocytosis by microglia in the hippocampus. This was accompanied by the restoration of serum levels of the microbiome-associated metabolites, acetate, and butanoic acid. On the other hand, SCFAs pretreatment was also found to improve cognitive impairment and reduce microglial activation and synapse engulfment in SD mice. Furthermore, our findings suggest that SCFAs prevent synapse loss via reducing synapse engulfment of microglia in microglia-neuron co-culture. We believe that further research on the potential benefits of *A. muciniphila* in preventing cognitive impairment caused by human sleep disorders is necessary. Finally, our findings highlight the essential role of SCFAs in maintaining microglial homeostasis against neural inflammatory stimulation. Disturbance of SCFAs level in the brain may be a key pathological process underlying multiple neurodegenerative disorders.

## Supplementary Material

Supplemental Material

## Data Availability

The data that support the findings of this study are openly available in the figshare, including 16S sequencing data (10.6084/m9.figshare.22180414) and metabolome data (10.6084/m9.figshare.22183780).
