# Supplementary material for: Akkermansia muciniphila supplementation prevents cognitive impairment in sleep-deprived mice by modulating microglial engulfment of synapses
Source: Gut Microbes. 2023 Sep 6;15(2):2252764. doi: 10.1080/19490976.2023.2252764 (PMC10484034; doi:10.1080/19490976.2023.2252764)
Supplement: Supplemental Material [file KGMI_A_2252764_SM2111.doc]

**Supplementary Materials**

**Methods**

**Supplementary Figure 1-11**

**Supplementary Table 1**

**Methods**

1. **Behavioral tests**

All behavioral tests were conducted during the light phase of the cycle between 08:00 and 16:00. Experimenters were blind to the condition when behavioral tests were carried out and analyzed. All the experimental mice are transferred to the behavior testing room 30 min before beginning the first trial to habituate to the condition of the behavior testing room. Behavioral tests were carried out in the following order: open-field test (OFT), elevated plus maze (EPM), novel object recognition (NOR), and Y-maze test.

**Open-field test:** The OF apparatus consisted of a 50 × 50 cm open arena with 30 cm high walls. The mouse was placed in the center of the arena and allowed to explore freely for 10 minutes. The total distance traveled in the inner (33 × 33 cm central area of the OF) and outer areas of the arena were recorded and analyzed by software (SMART 3.0; Panlab Harvard apparatus).^1^

**Elevated plus-maze test:** The apparatus used for the elevated plus maze test is in the configuration of a “+” and comprises two open arms (25 × 5 × 0.5 cm) across from each other and perpendicular to two closed arms (25 × 5 × 16 cm) with a center platform (5 × 5 × 0.5 cm). Mice were placed in the central square and moved freely for 5 minutes. Entry into an arm required the animal to enter that arm with all four paws. The number of entries (an entry is defined as the center of mass of the mouse entering the arm) into each arm and the time spent in the open arms are recorded and these measurements serve as an index of anxiety-like behavior.

**Novel object recognition test (NOR):** The novel object recognition test was performed in an open field arena. Stimuli consisted of plastic objects that varied in color and shape but had similar sizes. During training and test sessions, animals were placed in the center of the arena and exploratory behavior towards both objects was recorded for 10 min. The arena was cleaned with 20% ethanol between trials to eliminate olfactory cues. The training session was performed in the presence of two identical objects. For the test session, carried out one and a half hours after training, one of the two objects used in the training session was replaced by a novel object. Sniffing and touching the object were considered exploratory behavior, and the amount of time spent exploring each object was recorded by the software.^2^

1. **maze test:** The mouse was placed in the center of a standard Y maze (3 arms, 30 cm long, 120° apart) and freely explore the arms for 8 min. The number of all arm entries and alternations was recorded by the software.^3^
2. **Quantification of bacterial DNA in the feces of mouse**

Fecal samples were collected at four important time points to identify the effectiveness of antibiotic treatment, including [the](javascript:;) [first](javascript:;) [day](javascript:;) without any treatment, the 14 days after antibiotic treatment, the 7 days after sleep deprivation (SD) and the day after all of the behavioral tests finished. Oral administration of s to mice, collection of, DNA extraction and quantification of fecal bacteria by qPCR and immediately kept at -80°C until DNA extraction. Total genomic DNA was extracted from feces using the Stool DNA Kit (Omega, D4015, China) following the manufacturer’s instructions. DNA quantity was determined fluorometrically using the DeNovix-dsDNA Broad Range-Fluorescence Quantification Assay and the DeNovix DS-11+ Fluorometer (Wilmington, DE). DNA samples were diluted to a final concentration of 1 ng/µL using UltraPure DNase/RNase-Free Distilled Water (Invitrogen, Thermo Fisher Scientific, Waltham, MA). Real-time qPCR was performed on each sample in triplicate using 2 µL (2 ng of total DNA template) in combination with SYBR Green Premix (AG11701, Accurate Biology, China) following the manufacturer’s recommended guidelines. The forward HV3-16S primer 5′CCAGACTCCTACGGGAGGCAG-3′ and the reverse HV3-16S primer 5′-CGTATTACCGCGGCTGCTG-3′ (10µM) were added to the supermix. Real-time qPCR was carried out using a Bio-Rad CFX96 thermal cycler (Bio-Rad, Hercules, California, USA). Reaction mixtures (20 µL total volume) were held at 95°C for 30 s, followed by 40 cycles at 95°C for 5 s and 60°C for 30 s followed by a melting curve to verify nonspecific amplification. Plasmids verified to carry an HV3-16S insert for standard curve generation were constructed using a TOPO TA Cloning Kit (Invitrogen, Thermo Fisher Scientific, Waltham, MA) and gene inserts were obtained using genomic DNA from Escherichia coli strain ATCC 10536.

1. **Microbiota suspension preparation**

The microbiota suspension preparation was according to the methods described in the previous studies.^4, 5^ Briefly, feces pellets were collected from the SD mice 7 days after sleep deprivation and from control mice. Approximately 800-1000 mg of fresh feces (from 5-6 mice) were resuspended with a vortex in 6 ml of reduced PBS (PBS with 0.5 g/L cysteines and 0.2 g/L Na_2_S). The homogenate was centrifuged at 2,500 r.p.m. (500 g) for 5 min at 4°C. Supernatant fluid was diluted with an equal volume of 40% glycerin-PBS liquid and pack each tube 1.2 ml for 5 mice at a time, storing at -80°C until transplantation.

**The gene sequence of plasmid DNA**

TCCCCTACGGCTACCTTGTTACGACTTCATCCCAGTTACCAGTCTCACCTTAGGACCCTGCCTCCTTGCGGTTGGCTTCAGATACTTCGGGTGCGACCGGCTTCCATGATGTGACGGGCGGTGTGTACAAGACCCGGGAACGTATTCACGGCGCCGTAGCTGATGCGCCATTACTAGCGATTCCGGCTTCGTGTAGGCGGGTTGCAGCCTACAGTCCGAACTGGGCCCAGTTTTTAGGATTTCCTCCGCCTCGCGGCTTCGGCCCCCTCTGTACTGGGCATTGTAGTACGTGTGCAGCCCTGGGCATAAGGGCCATACTGACCTGACGTCGTCCCCACCTTCCTCCCAGTTGATCTGGGCAGTCTCGCCAGAGTCCCCACCTTCACGTGCTGGTAACTGGCAACAGGGGTTGCGCTCGTTGCTGGACTTAACCAAACATCTCACGACACGAGCTGACGACGGCCATGCAGCACCTGTGTAACGCCTCCGAAGAGTCGCATGCTTTCACATGTTGTTCATTACATGTCAAGCCCAGGTAAGGTTCTTCGCGTTGCATCGAATTAAGCCACATACTCCACCGCTTGTGCGGGTCCCCGTCAATTTCTTTGAGTTTTAATCTTGCGACCGTACTCCCCAGGCGGCACGCTTAACGCGTTAGCTCCGGCACGCAGGGGGTCGATTCCCCGCACACCAAGCGTGCACCGTTTACTGCCAGGACTACAGG

**Supplementary Figure**


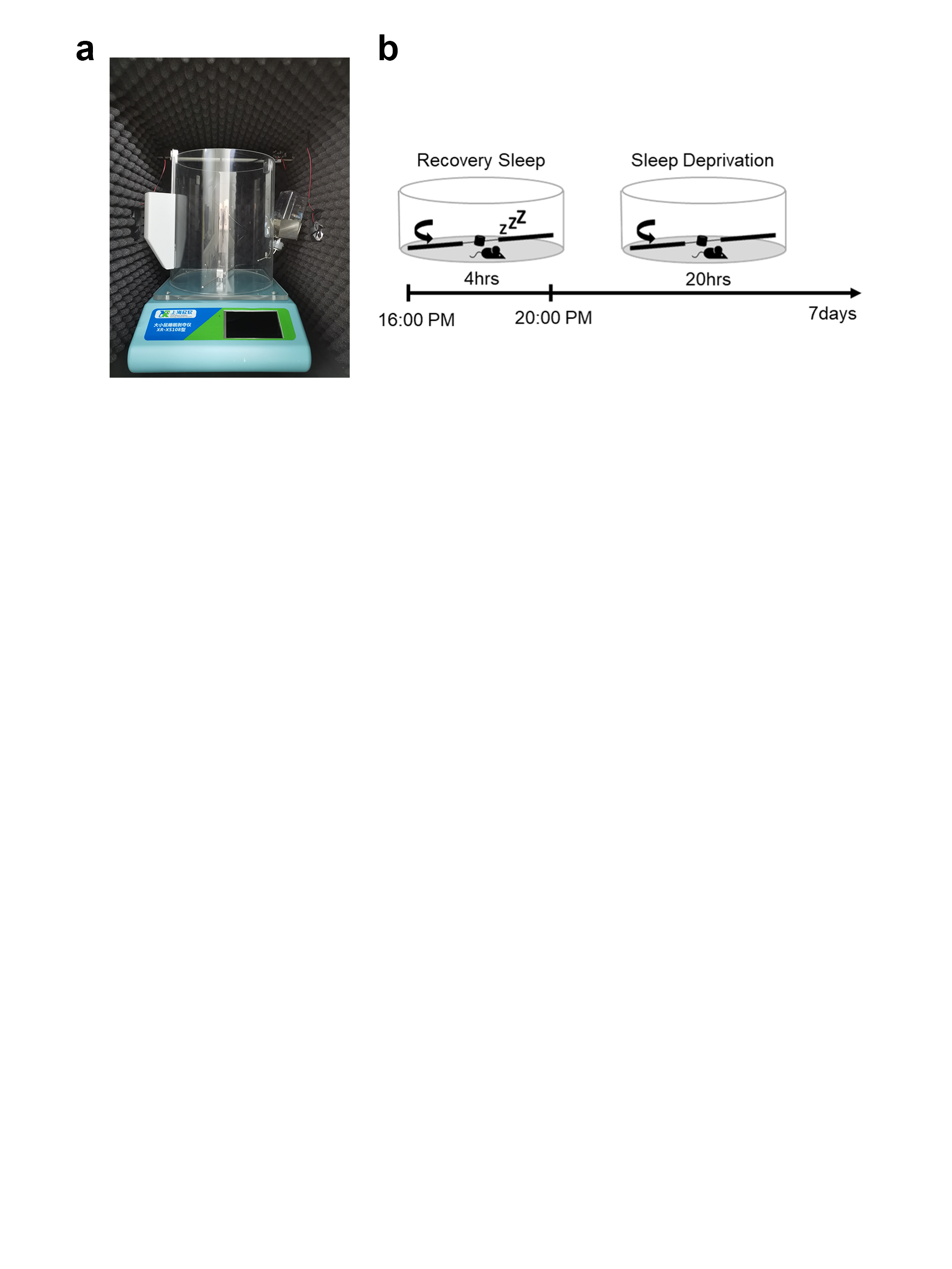


**Supplementary Figure 1. Schematic diagram of the sleep deprivation mouse model.** (a) Automatic SD system. (b) SD of the mice began from 20:00 to the next day16:00, after 20 hrs SD plus 4hrs recovery sleep. The total process lasted 7 days.


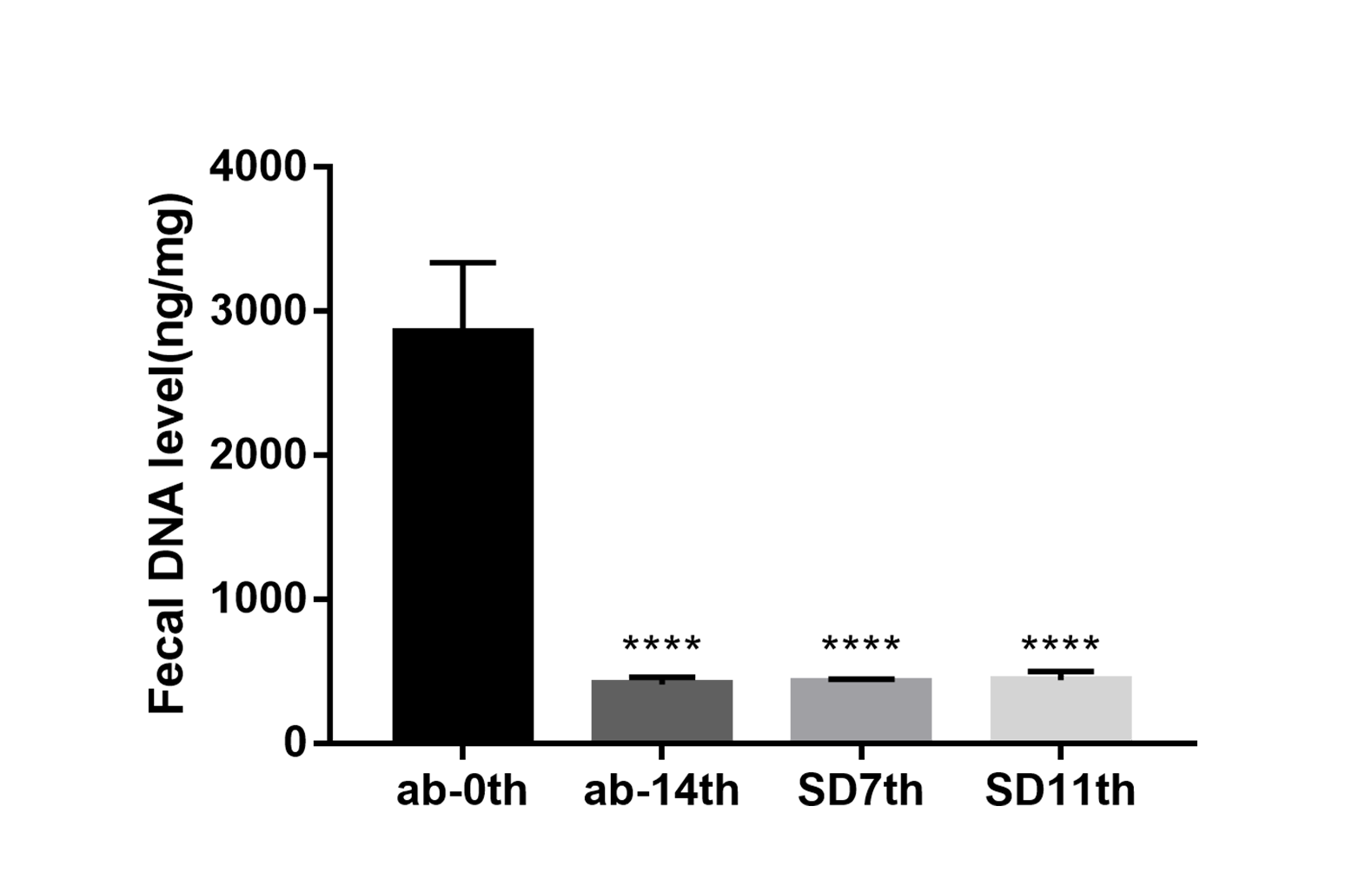

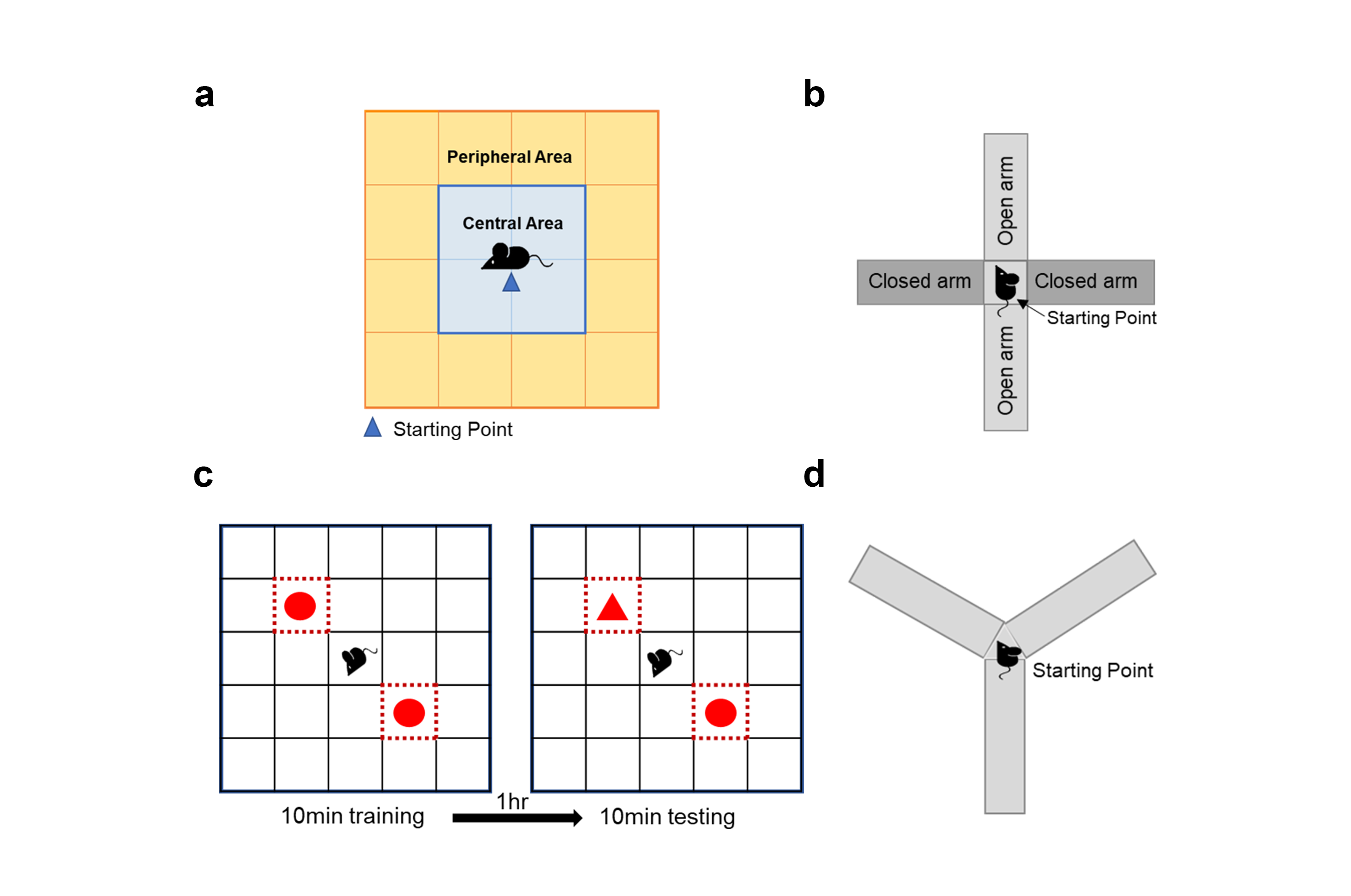
 **Supplementary Figure 2. Behavioral paradigms for studying anxiolytic properties and learning and memory in mice.** (a) open-field test (OFT), (b) elevated plus maze (EPM), (c) the novel object recognition (NOR), and (d) the Y-maze test.

**Supplementary Figure 3. The changes in the gut microbiome during the antibiotic treatment.** Antibiotic treatment for 14 days depleted the majority of gut bacteria and maintain the low level as demonstrated by fecal DNA concentration, n = 6-8 mice per group. (One-way ANOVA with Dunnett's multiple comparisons tests, *****P* < 0.0001).


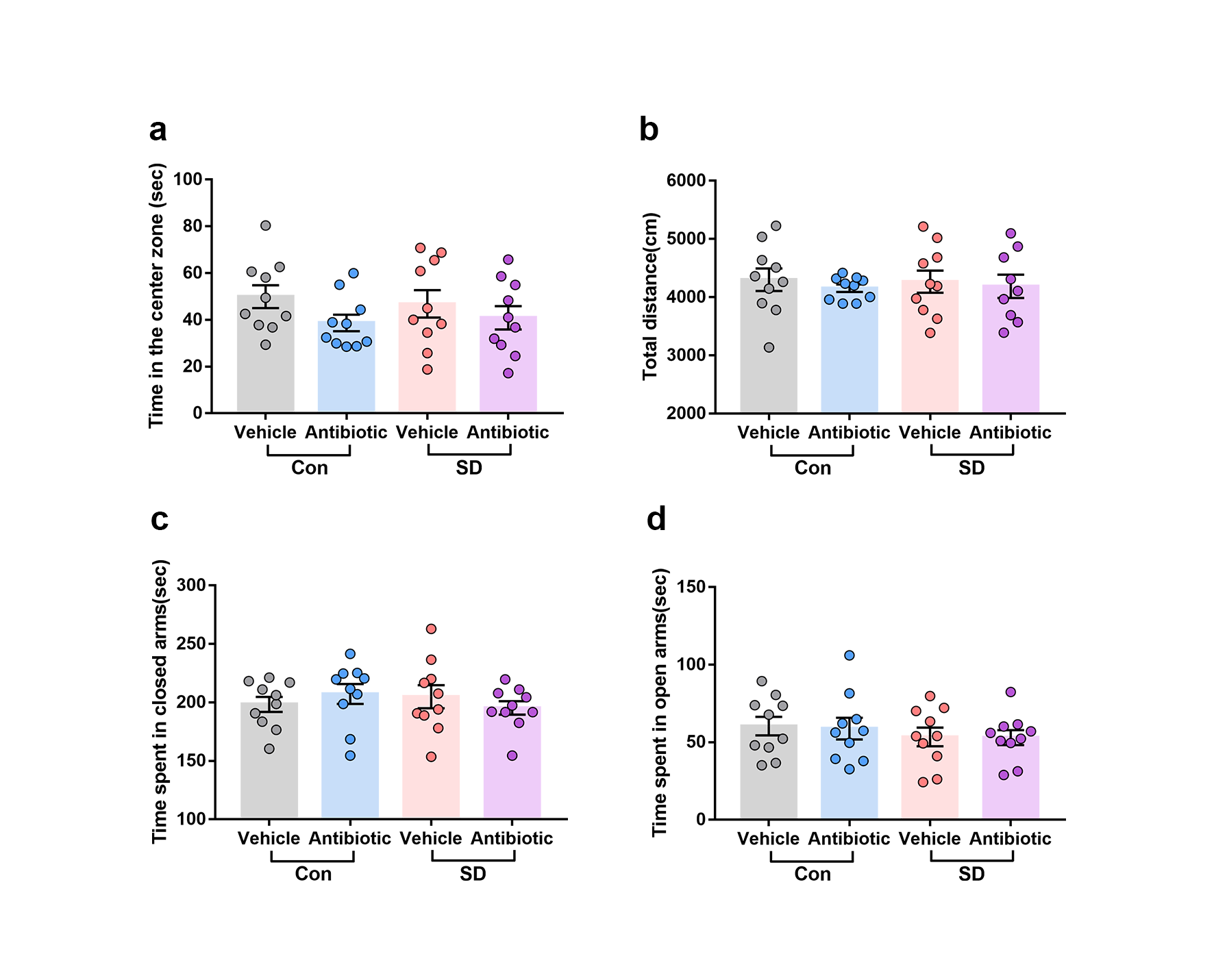


**Supplementary Figure 4. The effects of microbiome depletion on the host behavior in SD mice.** In the OFT test (a), time in the center zone (b) and the total distance (c) had no significant difference among the different groups. n = 10 mice per group, *P* > 0.05. In the EPM test (d), time in the closed arm (e) and time in open arms (f) had no significant difference among the different groups. n = 10 mice per group, *P* > 0.05. (One-way ANOVA with Tukey's multiple comparisons test).


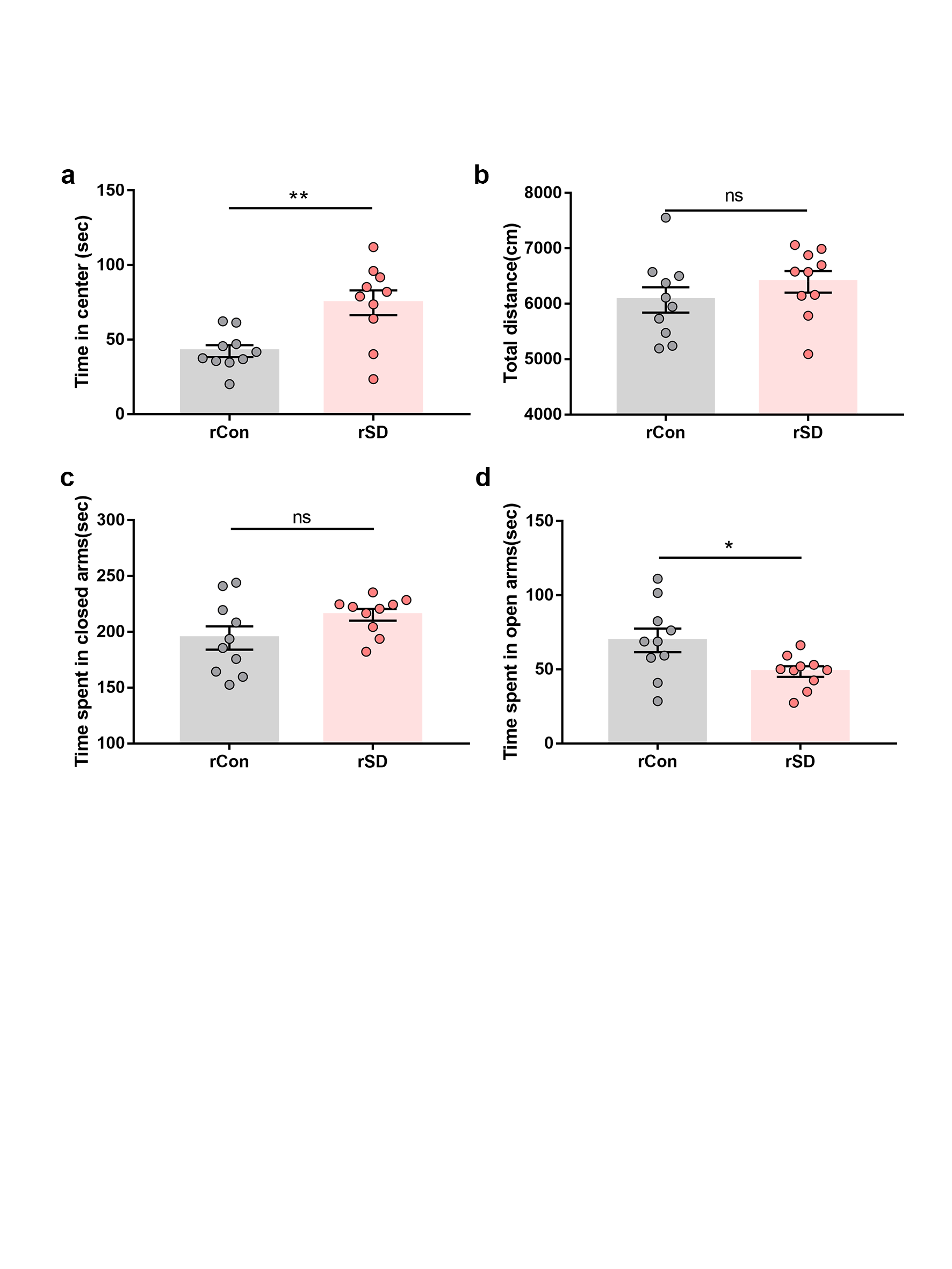


**Supplementary Figure 5. The effects of fecal microbiota transplantation (FMT) from SD mice in antibiotics-pretreated mice.** In the OFT test, time in the center zone (a) and the total distance (b) had no significant difference between the mice that received microbiota from SD mice and the mice that received microbiota from control ones. n = 10 mice per group, *P* > 0.05. In the EPM test, time in the closed arm (c) and time in open arms (d) had no significant difference between the mice that received microbiota from SD mice and the mice that received microbiota from control ones. n = 10 mice per group, *P* > 0.05. (Student’s *t*-test).


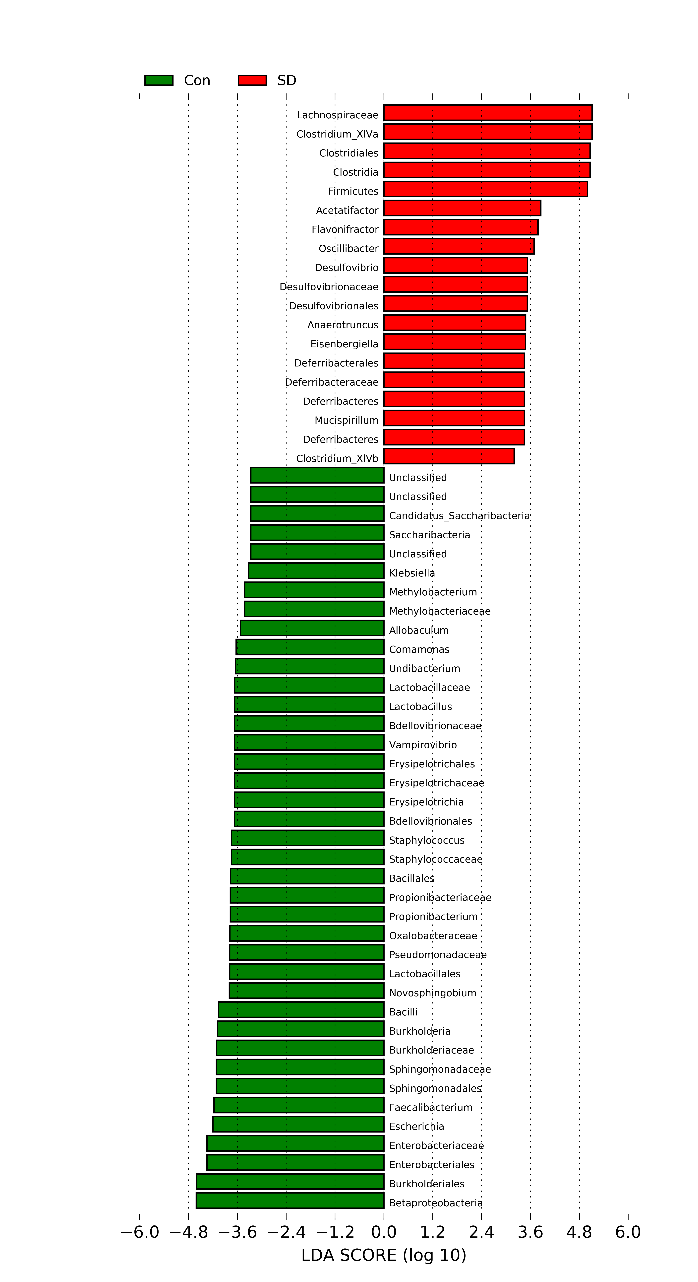


**Supplementary Figure 6.** **Supplementary Figure 6. LEfSe comparison of the gut microbiota between Con and SD.** The LDA score is greater than ±3.0. The length of the bar represents the LDA score.


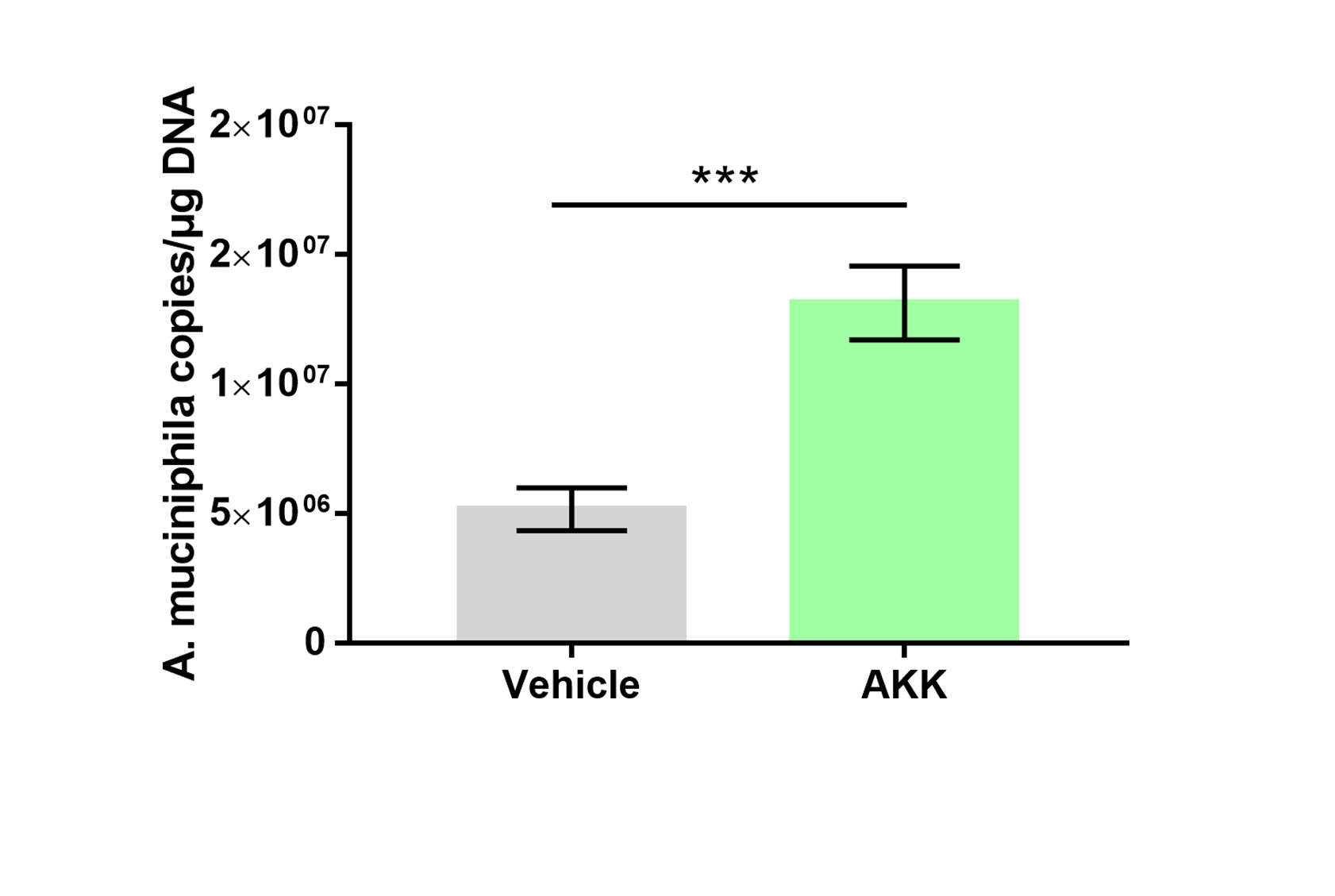


**Supplementary Figure 7. The changes in the abundance of *Akkermansia muciniphila* after bacterial transplantation.** DNA abundance of the 16S rRNA gene of *Akkermansia muciniphila* (*A. muciniphila*, AKK) was determined in feces from the mice colonized with *A. muciniphila* and vehicle-treated mice by q-PCR. Transplantation of *A. muciniphila* by oral gavage resulted in a significant increase in the concentration in feces the day after the behavioral test. n = 9 mice per group, ****P* < 0.001. (Student’s *t* test)





**Supplementary Figure 8.** **Metabolites differentiated between the SD/AKK and Con/V mice.** (a) Differentiation of metabolites between the SD/AKK and Con/V mice revealed by volcano plot. (b) The enrichment of representative metabolite in serum upon SD/AKK group.


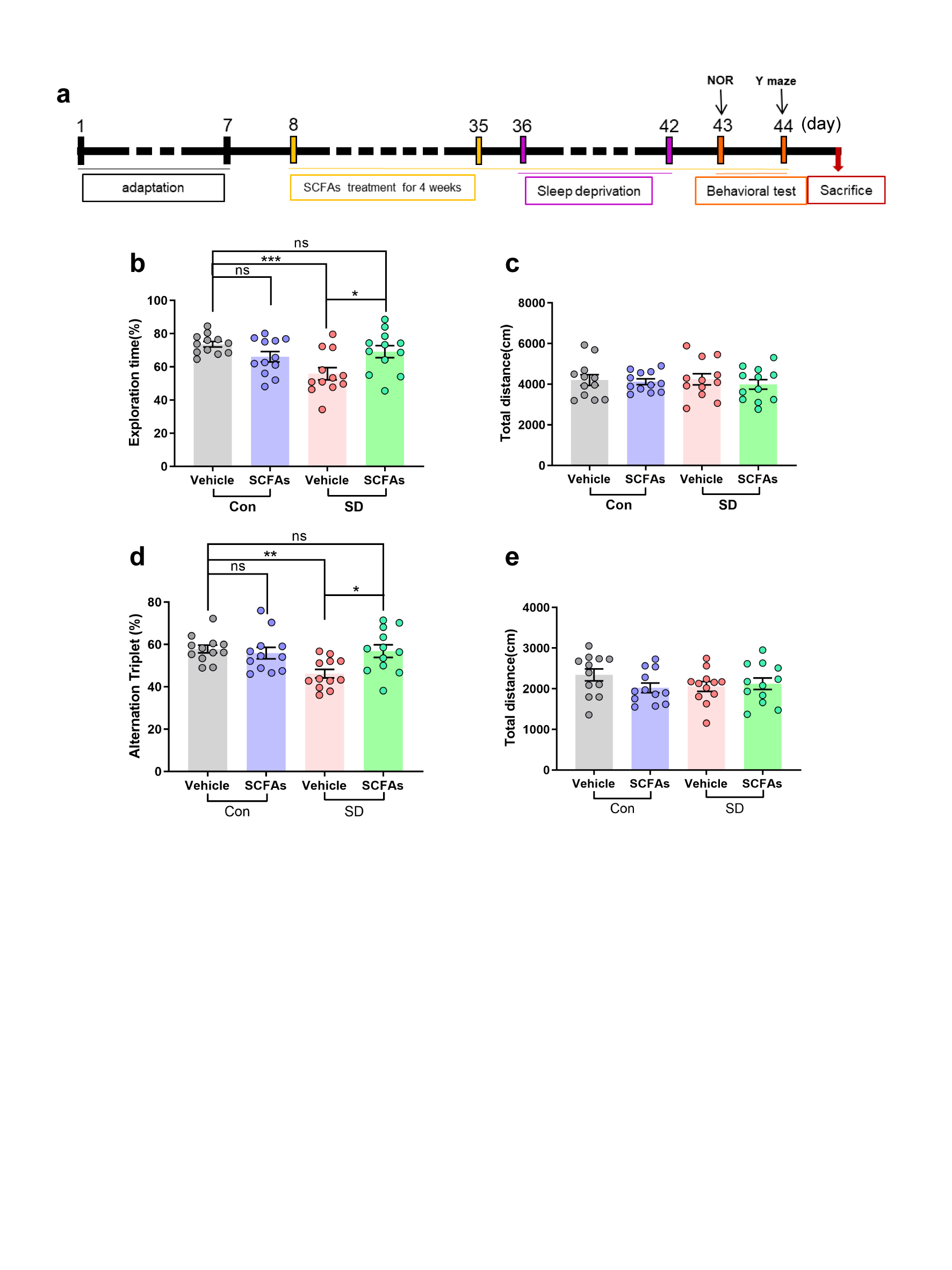


**Supplementary Figure 9. SCFAs treatment alleviated SD-induced cognitive impairment.** (a) Schematic of SCFAs pretreatment experiments. (b) Percentage of exploration time of mice from different groups in NOR test. (c) Total distance traveled among different groups in NOR test. n = 12 mice per group. (d) Spontaneous alternation index of mice from all groups in Y maze test. (e) Total distance traveled of mice in Y maze test. n = 12 mice per group. (One-way ANOVA with Tukey's multiple comparisons tests, **P* < 0.05, ***P* < 0.01, ****P* < 0.001, ns, no significant difference).


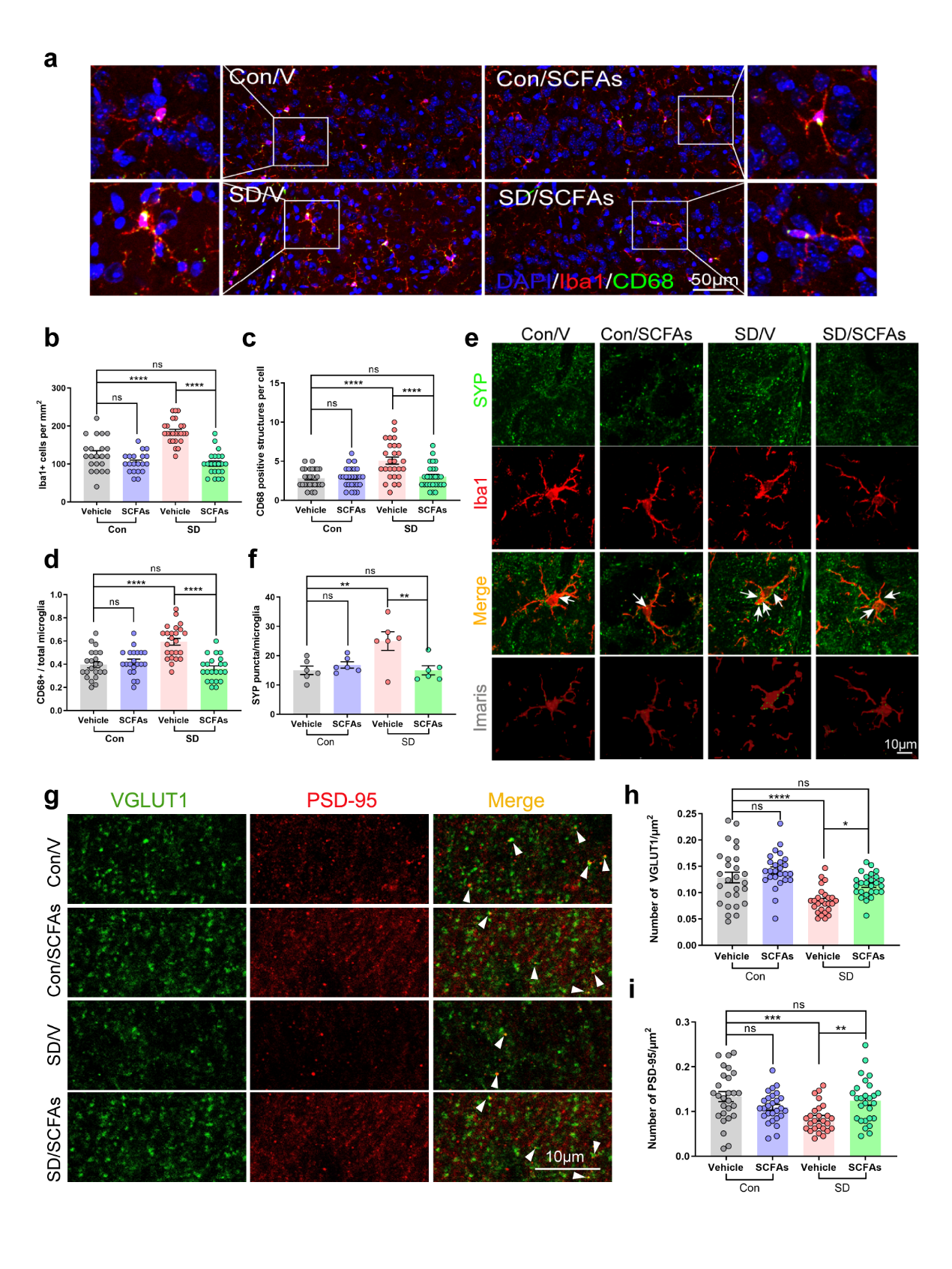


**Supplementary Figure 10. SCFAs inhibited microglial activation and synaptic engulfment in the hippocampus of SD mice.** (a) The representative immunofluorescence images of microglia (Iba1, red) and CD68 (green) double-staining of the hippocampus from each group. Scale bar = 50 μm. (b) The density of Iba1-positive cells in the hippocampus of each group. (c and d) Quantification of cells with CD68+ lysosomal accumulations. (e and f) Confocal/Imaris reconstruction images and quantification of SYP puncta within Iba1+ microglia in the hippocampus of each group (white arrows). n = 6 cells per group. (g) The representative images of VGLUT1 and PSD-95 in the dentate gyrus of each group (white arrows denote double-labeled puncta). Scale bar = 10 μm. (h) Quantification of VGLUT1 labeling. (i) Quantification of PSD-95 labeling. n = 26-27 areas per group. (One-way ANOVA with Tukey's multiple comparisons tests, **P* < 0.05, ***P* < 0.01, ****P* < 0.001, *****P* < 0.0001, ns, no significant difference).


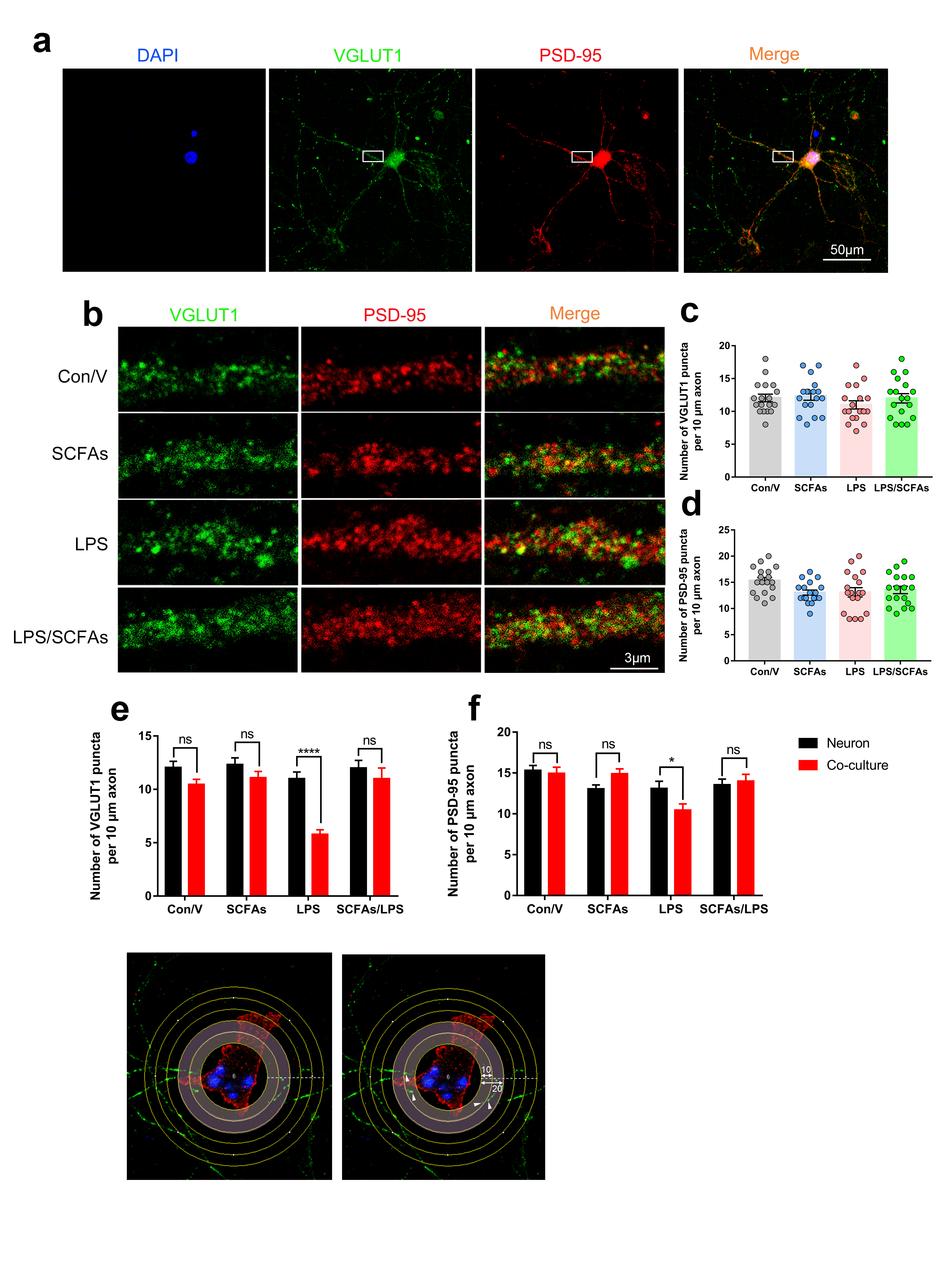


**Supplementary Figure 11.** **The effects of Short-chain fatty acids (SCFAs) on the synaptic engulfment of microglia *in vitro*.** (a and b) Representative images of VGLUT1(green), PSD-95 (red), and DAPI (blue) immunofluorescence in primary neurons. The segment for the dendrite was picked from each neuron and the locations for images taken were defined as 10 μm from the nucleus according to DAPI (indicated by the white square in (a). Scale bars, 50 µm (overview) and 3 µm (enlarge). (c and d) Quantification of VGLUT1 and PSD-95 densities in primary neurons without microglia. n = 18-20 fields per group. (One-way ANOVA with Tukey's multiple comparisons test). (e and f) Comparison of the VGLUT1 and PSD-95 densities between neurons from no microglia and neuron/microglia co-cultures. n = 18-20 fields per group. (Student’s *t* test, **P* < 0.05, *****P* < 0.0001, ns, no significant difference).

**Supplementary Table 1**

| **Primary antibodies** | | | | | |
| --- | --- | --- | --- | --- | --- |
| **Antigen** | **Species** | **Product(clone)** | **Manufacture** | **IF Dilution** | **WB Dilution** |
| PSD-95 | Mouse | sc-32291 | Santa Cruz | - | 1:500 |
| VGLUT 1 | Rabbit | sc-377425 | Santa Cruz | - | 1:100 |
| ACTB | Rabbit | AC026 | Abclonal | - | 1:1000 |
| PSD-95 | Rabbit | ab18258 | Abcam | 1:80 | - |
| VGLUT 1 | Guinea pig | 135 304 | Synaptic System | 1:200 | - |
| Iba1 | Rabbit | 019-19741 | Wako | 1:800 |  |
| CD68 | Rat | ab53444 | Invitrogen | 1:500 |  |
| mAb α-C1q | Mouse | ab71940 | Abcam | 1:50 |  |
| anti-synaptophysin-1 antibody | Mouse | 101011 | Synaptic System | 1:200 |  |
| **Secondary antibodies** | | | | | |
| Anti-Rabbit IgG | Mouse | 51275 | Cell Signaling Technology (CST) |  | 1:5000 |
| Anti-Mouse IgG | Goat | 4388 | proteintech |  | 1:3000 |
| Anti-rabbit IgG | Goat | 4413S | CST | 1:500 |  |
| Anti-Guinea Pig IgG | Donkey | 34506ES60 | Yeasen | 1:200 |  |
| Anti-Mouse IgG | Donkey | A-21203 | Invitrogen | 1:500 |  |
| Anti-Rat IgG | Goat | RS23240 | Immunoway | 1:200 |  |
| Anti-Mouse IgG | Donkey | A21202 | Invitrogen | 1:500 |  |

**References**

1. Tunc-Ozcan E, Peng CY, Zhu Y, Dunlop SR, Contractor A, Kessler JA. Activating newborn neurons suppresses depression and anxiety-like behaviors. Nature communications 2019; 10:3768. doi:10.1038/s41467-019-11641-8

2. Kincheski GC, Valentim IS, Clarke JR, Cozachenco D, Castelo-Branco MTL, Ramos-Lobo AM, et al. Chronic sleep restriction promotes brain inflammation and synapse loss, and potentiates memory impairment induced by amyloid-β oligomers in mice. Brain, behavior, and immunity 2017; 64:140-51. doi:10.1016/j.bbi.2017.04.007

3. Kosse C, Burdakov D. Natural hypothalamic circuit dynamics underlying object memorization. Nature communications 2019; 10:2505. doi:10.1038/s41467-019-10484-7

4. Bárcena C, Valdés-Mas R, Mayoral P, Garabaya C, Durand S, Rodríguez F, et al. Healthspan and lifespan extension by fecal microbiota transplantation into progeroid mice. Nature medicine 2019; 25:1234-42. doi:10.1038/s41591-019-0504-5

5. Zhu F, Guo R, Wang W, Ju Y, Wang Q, Ma Q, et al. Transplantation of microbiota from drug-free patients with schizophrenia causes schizophrenia-like abnormal behaviors and dysregulated kynurenine metabolism in mice. Molecular psychiatry 2020; 25:2905-18. doi:10.1038/s41380-019-0475-4
